# Supplementary material for: A 50 Hz magnetic field affects hemodynamics, ECG and vascular endothelial function in healthy adults: A pilot randomized controlled trial
Source: PLoS One. 2021 Aug 5;16(8):e0255242. doi: 10.1371/journal.pone.0255242 (PMC8341886; doi:10.1371/journal.pone.0255242)
Supplement: S1 File — (DOC) [file pone.0255242.s001.doc]

（Form1）

Receipt No.

Application form of the Human Research Ethics Committee of the Saitama University

To the Dean of the University

Date of submission: May 8^th^, 2017

Affiliation of applicant: Graduate School of Science and Engineering

Job title of applicant: Professor

Name of applicant: Keiichi Watanuki Seal

Please apply for the following research plan based on the provisions of Article15,Paragraph 1 of the Ethics Regulations on Human Research at Saitama University.

| 1. Type of   application | ☑New ・ ☐Modified | | | | | | | | |  |
| --- | --- | --- | --- | --- | --- | --- | --- | --- | --- | --- |
| 1. Title of   research | Evaluation of physiological effects of alternating magnetic field  exposure on human body | | | | | | | | |  |
| 1. Planned study   period | From the date of ethics committee approval to October, 31^st^ 2019 | | | | | | | | |  |
| 1. Investigators   (If there are  co-researchers  from other  institutions, they  should be listed) | Saitama University staffs and students (Affiliation, Job title: Name):  Graduate School of Science and Engineering, Professor;  Advanced Institute of Innovative Technology, Director:  Keiichi Watanuki  Graduate School of Science and Engineering, Master course student, 2nd year:  Akikatsu Fujimura  Graduate School of Science and Engineering, Master course student, 1st year:  Tsukasa Kondo  Advanced Institute of Innovative Technology, Visiting Ph.D. researcher:  Hideyuki Okano  ・Co-researchers  none | | | | | | | | |  |
| 1. Research place   (If research is  conducted at  another institution,  state whether there  is an ethics  committee at the  other institution  and review results) | ・Saitama University  Laboratory Building, 5th floor, No. 514 Laboratory, etc.  ・Other institutions  none | | | | | | | | |  |
| 1. Outline of   research | Study purpose, methods, and expected effects etc (State briefly):  We will evaluate the clinical effects of home-use electromagnetic therapy  devices (AC [50/60 Hz] magnetic therapy devices) that have been approved  by the Ministry of Health, Labor and Welfare in Japan for improving the  muscle stiffness and blood circulation in the exposed regions, using the latest  multiple biomonitoring systems, e.g., muscle and peripheral blood flow, near-  infrared spectroscopy (NIRS) signal, endothelial function (flow mediated  dilation [FMD]), electrocardiogram (ECG), electromyogram (EMG), muscle  hardness, etc. Here we have developed methods to objectively evaluate the  physiological responses of users (including potential users) to magnetic  fields.  By visualizing and quantifying the conditions of muscles and blood vessels  in particular, the reliable scientific evidence on AC magnetic therapy can be  obtained, and this will contribute to the development of treatment methods  and therapy devices that lead to improvement of various symptoms and  recovery from fatigue. It is considered that more appropriate and effective  treatment information (information on proper dosage, etc.) can be provided.  be provided. | | | | | | | | |  |
|  | Category of  research | | ☐Human genome / gene analysis research | | | | | | |  |
|  |  |  | ☐Clinical medical research | | | | | | |  |
|  |  |  | ☑Other clinical research | | | | | | |  |
|  | Handling of  Personal  information | | ☐No | | ☑Yes→Describe below. | | | | |  |
|  |  |  | Name, age, gender, height, weight, body temperature and  blood pressure | | | | | | |  |
|  | Examination  of specimens  from the  human body | | ☑No | | Yes→Describe below. | | | | |  |
|  |  |  |  |  |  |  |  |  |  |  |
|  | Invasiveness | | ☑No | | ☐Major→ | | | Type of invasiveness  ☐Physically／☐Mentally | |  |
|  |  |  |  |  | ☐Minor→ | | |  |  |  |
|  | Intervention | | ☐No | | ☑Yes | | | | |  |
| 1. Research   participants | Age group, gender, expected number of participants, etc.:  Healthy volunteers (including athletes), both genders, 20–90 years old, about  100 individuals  Inclusion criteria:  During the study period, participants have not used any form of physical  therapy and have not taking any vasoactive medication. Subjects’ body  temperature and systolic and diastolic blood pressures were within normal  ranges. We obtain a written informed consent for subjects’ participation.  Enrollment methods (Describe exclusion criteria if any):  Study subjects in their 20s years will be recruited from students at Saitama  University, and subjects aged 20–90 years will be recruited from the general  public.  Exclusion criteria:  The above inclusion criteria are not met during the study period.  Honorarium / transportation fee etc.:  Participants recruited from the general public in 30–90 years will be  paid fees according to the experiment time. | | | | | | | | |  |
|  | Under 20 years | | | ☑No | | | ☐Yes→  Legally authorized representative Yes/No | |  | |
|  | Good judgment  and awareness | | | ☑Yes | | | ☐No→  Legally authorized representative Yes/No | |  | |
|  | Criteria for legally authorized representative if any: | | | | | | | | |  |
|  | Disease /  Disability | | | ☑No | | | ☐Yes→Describe below. | |  | |
|  | Consideration for subjects with diseases and disabilities if any: | | | | | | | | |  |
| 1. Means to   explain to  research  participants and  to obtain  informed consent  (Details of  explanation /  consent, e.g.,  voluntary  participation in  research) | We explain to participants (subjects) in advance using study documents．  We get the following informed consent from all participants:  Participation in the experiment is entirely voluntary, and if you feel  unwell, you can cancel participation at any time during the experiment.  The acquired experimental data is statistically analyzed and no  individual is identified. Even after the end of the experiment, it is  possible to cancel participation in the experiment by request and discard the  experiment data. | | | | | | | | |  |
| 1. Experimental   protocol  (The list of  specific  descriptions) | 1. We show an AC magnetic therapy device to subjects.  2. The subjects participate in the trials of an AC magnetic field (MF)  exposure (either MF exposure or sham exposure) in a randomized double-  blind manner (a method unknown to participants and technicians). At that  time, the assignment of MF exposure or sham exposure is performed by a  study administrator (except for participants and technicians). This is to  prevent the effects of placebo and observer bias.  3. We perform MF or sham exposure and monitor physiological parameters  (blood flow, NIRS, FMD, ECG, EMG, etc.) before, during, after (at fixed  time intervals) or continuously.  4). We perform the above trials by monitoring the parameters of subjects in  their sitting or supine position.  5. As an optional test, physiological parameters are measured before and  after the experimental task combining the muscle fatigue test (training with a  dumbbell, treadmill exercise, etc.) with magnetic therapy. | | | | | | | | |  |
| 1. Publication   methods of  study results  (Including  consideration  for personal  information) | In addition to submitting to domestic and foreign academic journals,  we will present at domestic and international academic conferences.  At the time of publication, no personally identifiable information is  presented, and only the summary results after statistical analysis are  described. | | | | | | | | |  |
| 1. Means to   protect personal  information and  experimental data | Data  anonymization | | | ☐Unlinkable anonymized data is saved. | | | | |  | |
|  |  |  |  | ☑Linkable anonymized data is saved together with  separate correspondence tables. | | | | |  | |
|  |  |  |  | ☐Others ( ) | | | | |  | |
|  | Disposal method | | | ☑After storing for a certain period of time based on  internal regulations, we anonymize the data connection  and discard the data. | | | | |  | |
|  |  |  |  | ☐Others ( ) | | | | |  | |
|  | Means to provideto other  institutions  (if applicable) | | | ☐We don’t present separate correspondence  tables. Instead, we provide unlinkable anonymized  data. | | | | |  | |
|  |  |  |  | ☐Others ( ) | | | | |  | |
|  | Specific storage / disposal / provision methods and storage period:  First, the data files accumulated in the experiments are stored in a locked  environment using a storage medium physically disconnected from the  internet. Next, data files stored on digital media are discarded after the  media is physically destroyed and rendered unreadable. Data recorded on  paper is discarded after shredding using a shredder. | | | | | | | | |  |
| 1. Potential   participant’s  disadvantages  and risks,  methods for  protecting  human rights  (Describe  specifically  means to deal  with each case  and criteria for  discontinuing  research) | The AC magnetic therapy device is considered to be a safe treatment  device because there have been no reports of adverse health effects such  as side effects and adverse events. Since the subjects are healthy  volunteers, there are no items that refer to “Precautions for Use” or  “Contraindications / Prohibitions” when conducting this study.  The measurement of vascular endothelial function, i.e., flow-mediated  dilation (FMD) used in this study, is a non-invasive measurement method.  At the time of the measurement, a cuff is wrapped around the forearm in  the supine position and the blood is fed for 5 min. There is a possibility  that pain and numbness may occur due to reduced blood transport (in an  outsourced survey by a manufacturer of a publicly available testing device,  about 20% of subjects had numbness but had no pain at all). We conduct  experiments after confirming the physical condition in advance, and always  observe the subjects during the experiments. If any physical condition is  observed, stop the experiments and try to avoid any danger.  The experimental data should be under high security control. When  analyzing the data, avoid installing software with unstable security level to  prevent the leakage of digital data, and make the best use of antivirus  software.  Inquiries and complaints to the technicians and researchers will be set  up, and a prompt response system will be established for handling  complaints. All experiments will be conducted with due consideration to  the health and mental aspects of the subjects. Also, in the operation of  various experimental devices, the technicians and researchers should be  very careful not to cause accidents due to human error by carrying out  sufficient training. | | | | | | | | |  |
| 13. Attachments | ☑Study documents for participants ☑Informed consent ☑Others  (Consent withdrawal form) | | | | | | | | |  |
|  | Reasons for not attaching: | | | | | | | | |  |
| 14. Main funding | ☐Operating expenses ☐Grant-in-Aid for Scientific Research  (Category: Principal investigator: )  ☑Others (Joint research expenses) | | | | | | | | |  |
| 15. Other remarks |  | | | | | | | | |  |
|  | | Confirmation of  department head | | | | Date: | | | | |

Informed consent form for study participation

Notice to participants Year , month , day

Request of participation for

“Evaluation of the physiological effects of AC magnetic field exposure on humans”

Please read this manual before starting the clinical study, and if you would like to cooperate, please sign the participation agreement.

Purpose of research

Currently, there are many reports on the effectiveness and safety of exposing the human body to AC magnetic fields. For example, there are some reports that AC magnetic fields have fatigue recovery effect as well as skin temperature increase effect. However, detailed examination has not been conducted on what kind of physiological responses the magnetic fields could induce.

The main purpose of this study is to evaluate the effects of exposure to an AC magnetic field on blood flow velocity, tissue oxygen concentration, and vascular endothelial function by combination with digital color Doppler ultrasonography in the forearm (ulnar artery), functional near-infrared spectroscopy (fNIRS) in the forearm (flexor muscles), electrocardiogram (ECG), and brachial artery flow-mediated dilation (FMD) test.

Method

Blood flow velocity

1. Sit in the designated chair and place your left arm on the magnetic field exposure device.

2. After resting for 5 min, the technician presses the probe against the forearm and measures blood flow velocity.

3. After resting, the blood flow velocity is measured at 5-min intervals, and one testing trial is completed when all 6 serial measurements are accomplished. The time required is about 40 min per trial.

NIRS

1. Sit in the designated chair and attach the 2-channel pocket NIRS sensors to the two parts of the forearm flexor muscles.

2. Hold a 3 kg dumbbell in your left hand (in the case of a right-handed person) and do dumbbell wrist curl training for 5 min.

3. After that, keep your left arm on the magnetic field exposure device for 25 min. The measurements during that time are continuous and automatic.

ECG

1. Sit in the designated chair and attach the magnetic field exposure device to the back side of the neck.

2. A telemetry ECG probe is attached to the chest and rested for 5 min.

3. After that, keep the same posture for 25 min. The measurements during that time are continuous and automatic.

FMD

1. Lying on the designated bed in the supine position, place the left arm on the magnetic field exposure device.

2. A blood pressure cuff is attached to the left forearm, the probe is fixed to the upper arm, and after resting for 5 min, FMD is measured.

3. FMD is measured twice in one trial. One measurement time is about 10 min.

4. The second measurement of FMD will be performed 30 min after the first measurement. It takes about 1 hour to complete one trial in the supine position.

Each trial for each participant was performed twice on different days after a washout period of at least two days.

Handling of personal information and data

The only way to obtain personally identifiable personal information in this study is to sign the consent form. Data and personal information that cannot identify individuals will not be used for purposes other than research. Since the data is numbered and anonymized, personal information will be kept confidential even when the research is presented through specialized academic societies, academic journals, on-campus research groups, etc. We take all possible measures to store data and do not leak it to the outside. This stored data will be destroyed within 5 years after the end of the study.

The rights of the test subjects

It is up to you to decide whether or not to participate in this study. In addition, even if you agree once, you can revoke your consent, and there is no disadvantage.

In addition to discarding the anonymization number, the data and analysis results obtained so far will be discarded and will not be used for further research. However, please note that this data cannot be destroyed if there are published analysis results at the time of the cancellation request.

Benefits and disadvantages of participating in this study

There is no cost burden for participating in this study. You will not be penalized if you do not participate. For current students, it has nothing to do with the grade evaluation of the courses taken. We will do it with safety in mind within the scope of previous academic research examples. If something goes wrong, the experiment will be canceled, so please inform the technicians or investigator immediately even during the experiment.

Contact information

If you feel that the rights of those participating in this study are not being protected, or if you would like opinions or information other than the person in charge, please contact us at the following contact information.

(Contact information)

Person in charge: Research Cooperation Division Ishikawa and Saito

External line +81-48-858-9742，Internal line 3586，E-mail: kshinkou@gr.saitama-u.ac.jp

For the progress and results of this study, please check with the technicians/investigator or the supervisor of the study.

If you have any questions, please do not hesitate to ask us more information.

We deeply appreciate your understanding and cooperation in this study.

(Technicians and investigator）

Graduate School of Science and Engineering, Saitama University

Akikatsu Fujimura, Tsukasa Kondo

Advanced Institute of Innovative Technology, Saitama University

Hideyuki Okano

(Supervisor)

Advanced Institute of Innovative Technology, Saitama University

Hideyuki Okano

Informed consent form for study participation

“Evaluation of the physiological effects of AC magnetic field exposure on humans”

≪Checklist which you received explanation and understood.≫

□ Purpose of this study

□ Method

□ Handling of personal information and data

□ The rights of the test subjects

□ Benefits and disadvantages of participating in the experiment

□ Contact information for inquiries, complaints, etc.

□ Name, affiliation, and job title of the principal investigator

I understand the above explanation and agree to participate in this study.

Year , month , day

Affiliation: ＿＿＿＿＿＿＿＿＿＿＿＿＿＿＿＿＿＿

Full name: ＿＿＿＿＿＿＿＿＿＿＿＿＿＿＿＿＿＿

**Experimental protocol**

**Randomization**

All right-handed participants were divided evenly into two conditions, a real magnetic field (MF) (A) and a sham (B) exposures, with an allocation ratio of 1:1 after block randomization with a permuted block size of 2 according to computer-generated random numbers by an administrative controller/investigator. Thus, the allocation of which condition would receive either a real MF (A) or a sham (B) exposure for the first time was randomly generated. When considering the case of the flow chart of this study, 2-treatment and 4-period crossover design (2 × 4 crossover sequence) was done; ABAB or BABA.

**Blindness**

The allocation was blinded to both the participants and the technicians. Real MF and sham control exposures were also blinded to both of them except for one investigator who was regarded as an administrative controller/investigator of an MF exposure device who did not have any contact with the participants and technicians. The operation switch was turned on and off remotely by the investigator using an extension cord. Technicians were not allowed to check the switch of the MF exposure device. Participants were not informed as to when the MF exposure device was switched on or off.

**Statistical analyses**

For all subjects, the following measurement items were measured in MF and sham exposures for 2 trials each on different days, and the measured values of MF and sham exposures were compared between them, and the following significant difference tests were performed. A two-way repeated-measures ANOVA was calculated with the factors “Time” and “Condition”. *Post hoc* analysis of differences between the MF and sham exposures was made with the Student’s *t*-test or the Wilcoxon rank-sum test (between conditions), and analysis of within the same exposure was performed with the paired *t*-test or the Wilcoxon signed-rank test (within a condition) with two-sided tests, according to the normality of samples’ distribution verified with the Shapiro-Wilk test. For all comparisons, *p* < 0.05 was considered significant.

**Blood flow velocity**

The measurement of blood flow velocity was evaluated by the peak systolic blood flow velocity (PSV) by the ultrasonic Doppler method using ultrasonic echo. The results of PSV measurement are shown in the display monitor of an ultrasonic echo imaging device in the below figure. The ultrasonic Doppler method is a method of finding the moving speed of red blood cells, i.e., the blood flow velocity, using the Doppler effect in which the frequency of sound changes when the irradiated ultrasonic waves are reflected by the red blood cells moving in the blood vessels. Here, assuming that the ultrasonic incident angle *θ* and frequency *f*_0_ emitted from an ultrasound probe, the ultrasonic frequency *f*_d_ reflected by red blood cells, and the speed of sound *C* in the living body, the blood flow velocity *V* can be obtained as shown in equation (1):


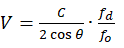
 (1)

where the ultrasonic incident angle *θ* was set within the range of 45–60°, which is considered to have no effect on PSV measurement in this study. The blood flow in the blood vessels has been found to be hydrodynamically laminar. The sample volume required to measure the blood flow velocity was set to about 1/2 to 2/3 of the inner diameter of the blood vessel. In the case of the tortuous blood vessels, it was considered difficult to set the sample volume at the center of the blood vessel. Therefore, the tortuous blood vessels were not measured. In addition, since the blood vessel diameter varies not only over time but also depending on the measuring site, when the measurement site is not the same site each time, the variation in blood flow velocity may increase further as measurement error. Therefore, in order to be able to measure at the same site as much as possible each time, the skin surface of the first measurement site was marked with an oil-based black pen, and then the second and subsequent measurements were repeated.


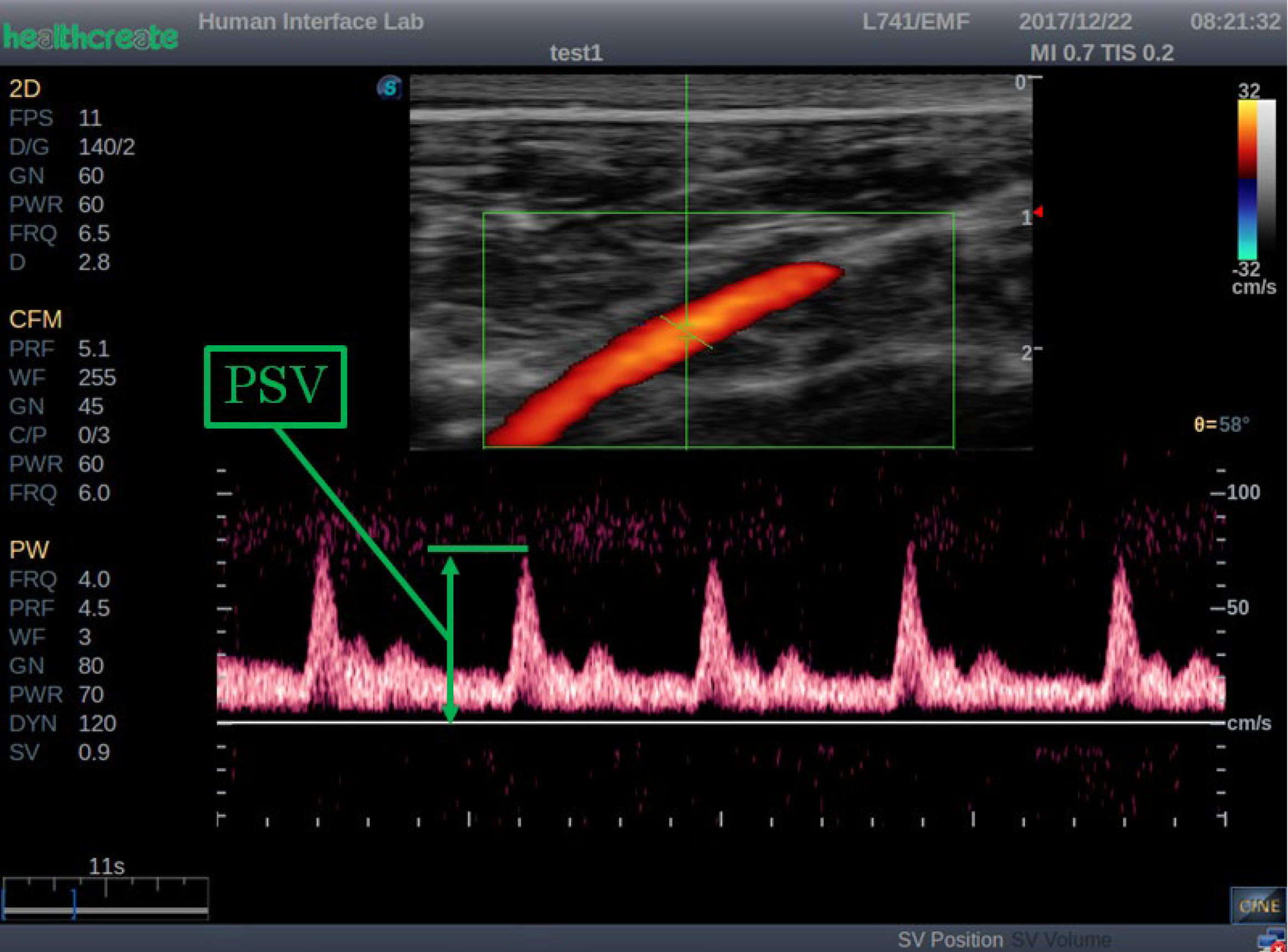


**The results of PSV measurement**

An ultrasonic system with a portable digital color Doppler ultrasound system (JS2 with a linear ultrasound probe L741, 5–12 MHz, Medicare Co., Ltd., Shenzhen, China) was used for PSV measurement. As shown in the figure below, the blood flow velocity of the ulnar artery was measured for 25 min at 5-min intervals with the ultrasound probe in contact with the skin surface of the ventral side of the left forearm. Each subject kept the left palm up and the left arm bent 120–140°, placed the forearm on an MF exposure device, rested for at least 5 min, and then exposed to MF or sham for 15 min. The site of MF exposure is the forearm, upper arm, or neck as shown in the figure below. The MF-exposed side of the forearm and upper arm was to the dorsal side of the arm, and that of the neck was to the back side of the neck. In each case, the forearm was placed on the MF exposure device. When the upper arm and neck were exposed to the MF, an additional device different from the MF exposure device used for the forearm was used, and the forearm was not exposed at the same time.


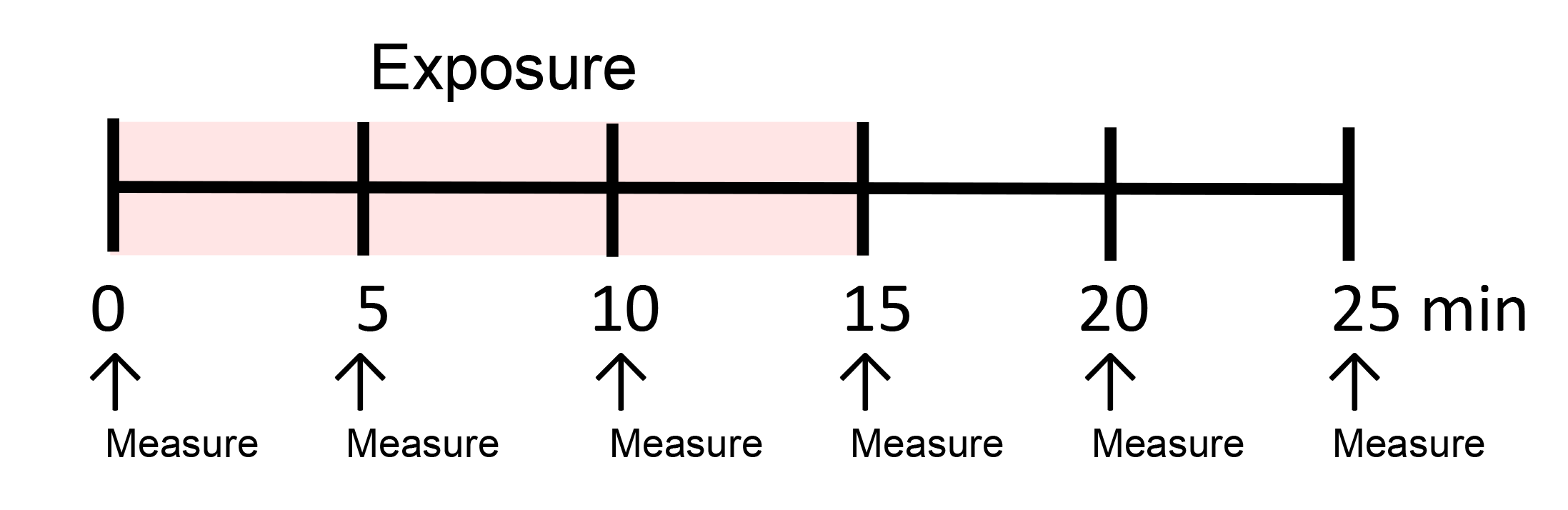


**Experimental time protocol**


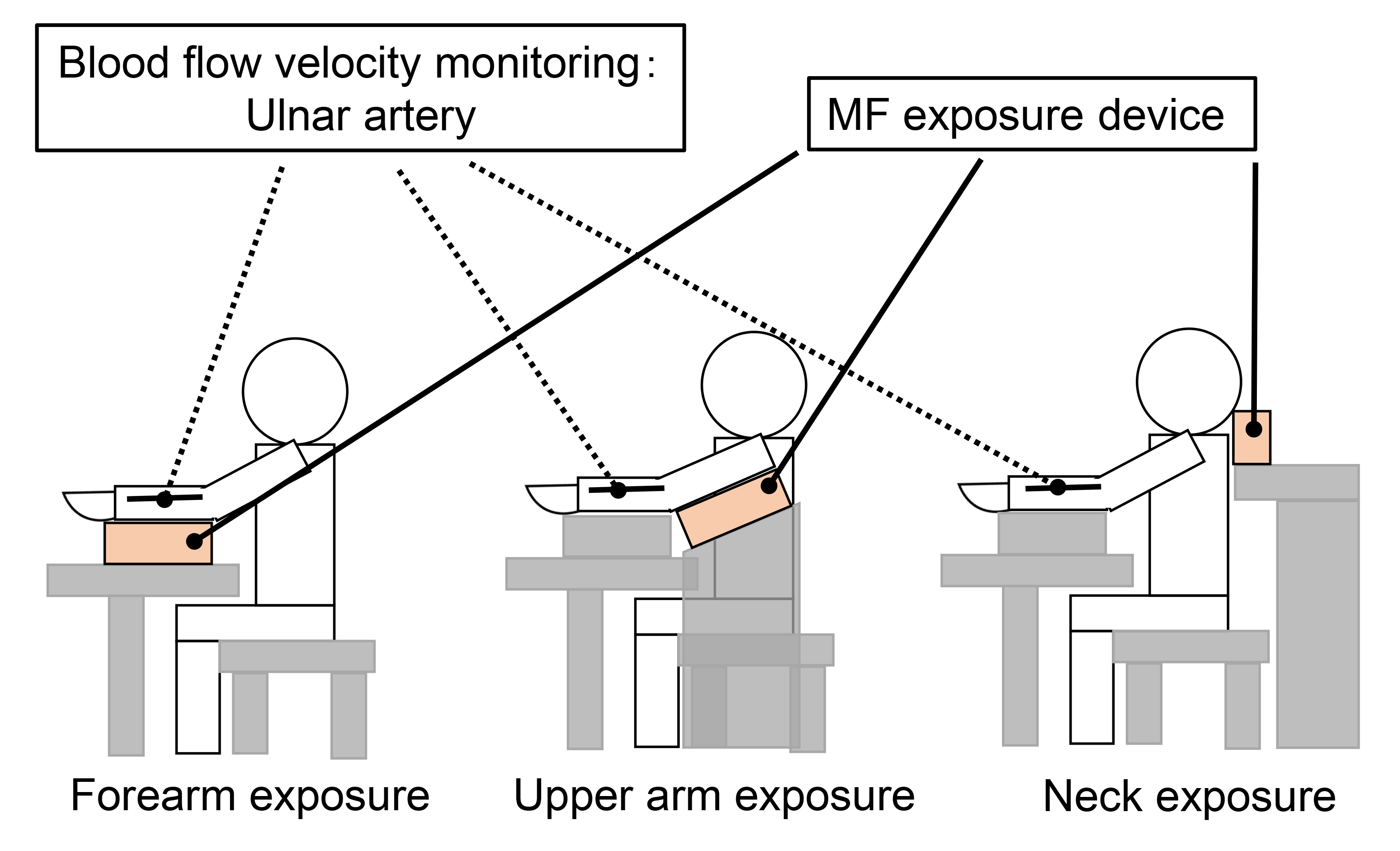


**Blood pressure and heart rate measurements**

Blood pressure and heart rate were measured by the same method as in the forearm exposure experiment. As shown in the figure below, measurements were taken at 10-min intervals using a digital sphygmomanometer with an upper arm cuff (DSK-1051J, NISSEI Co., Ltd., Tokyo, Japan) before, during, and after exposure.


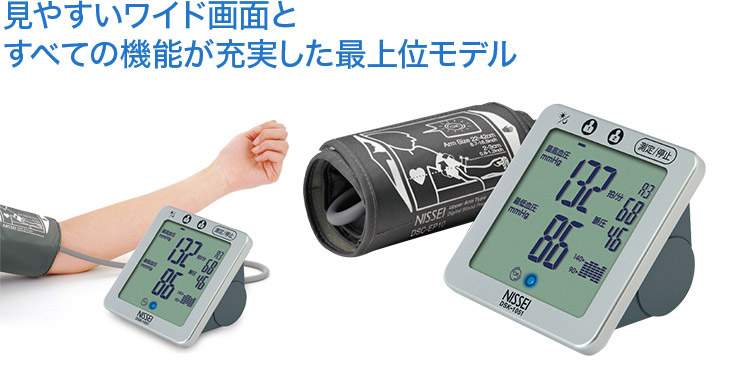


**Upper arm digital sphygmomanometer**


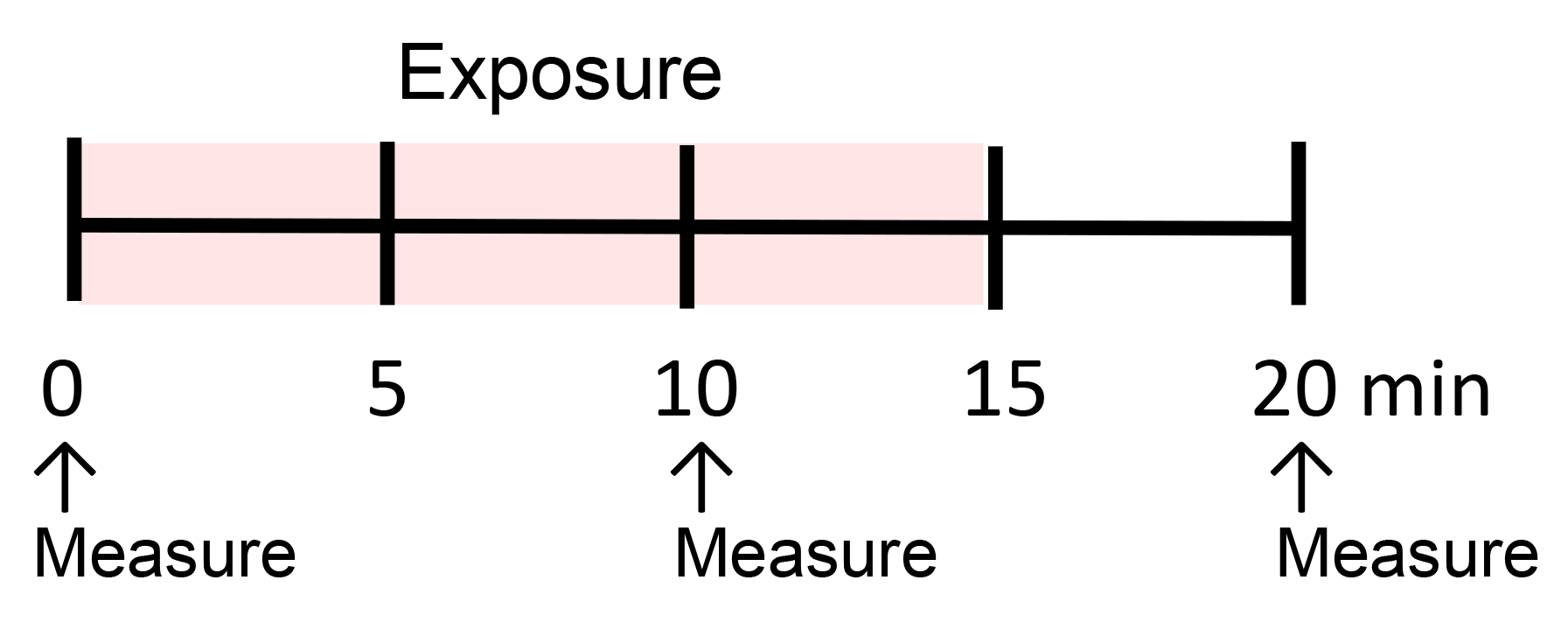


**Experimental time protocol**

**Selected muscle loading exercise**

In order to select the most suitable method for muscle loading exercise (training) at the wrist flexor muscles in the left forearm, evaluation was performed by the following three methods. (1) Dumbbell wrist curl was used to train the forearm flexor muscles. This is a method of fixing with the front of the forearm facing up using a 3 kg dumbbell (SINTEX Chrome array 3kg STW021, Sinwa Enterprise, Osaka, Japan) and raising and lowering the wrist. (2) Reverse wrist curl was performed for the training of the forearm extensor muscles. This is a method of fixing the forearm with the back surface facing up and raising and lowering the wrist. (3) As training for the brachioradialis, hammer curl was performed. This is a method in which the arm is straight down from the shoulder, the dumbbell is held so that the palm faces oneself, and the elbow is bent and stretched. As a result, when the dumbbell wrist curl was performed, the increase rate of muscle hardness was the largest at the wrist flexor muscles before and after the training compared to the other two training methods, so dumbbell wrist curl training was selected.

**Wrist curl training**

As shown in the figure below, each participant performed the wrist curl exercise for 5 min in a chair sitting position. The participant held the dumbbell, kept the forearm stationary, turned the palm up, and raised the wrist freely, then raised the dumbbell to the dorsiflexion position of the wrist joint in 1 s. Thereafter, the dumbbell was lowered to the maximum range of the wrist flexion from the dorsiflexion position in 2 s. It took 3 s to perform 1 cycle. The participant conducted 20 repetition cycles in 1 min and after that took a rest for 1 min. Thus, a 5-min exercise consisted of 3 sets of 20 repetition cycles with a 1-min interval between sets.


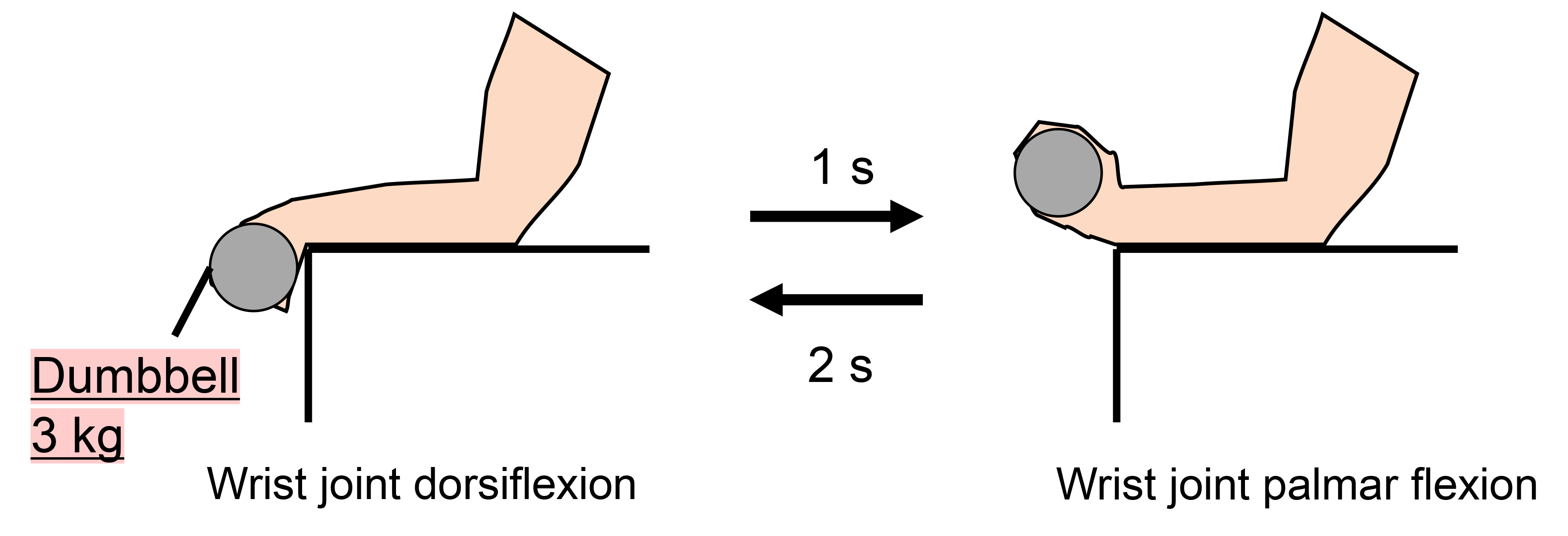


**Wrist curl training**


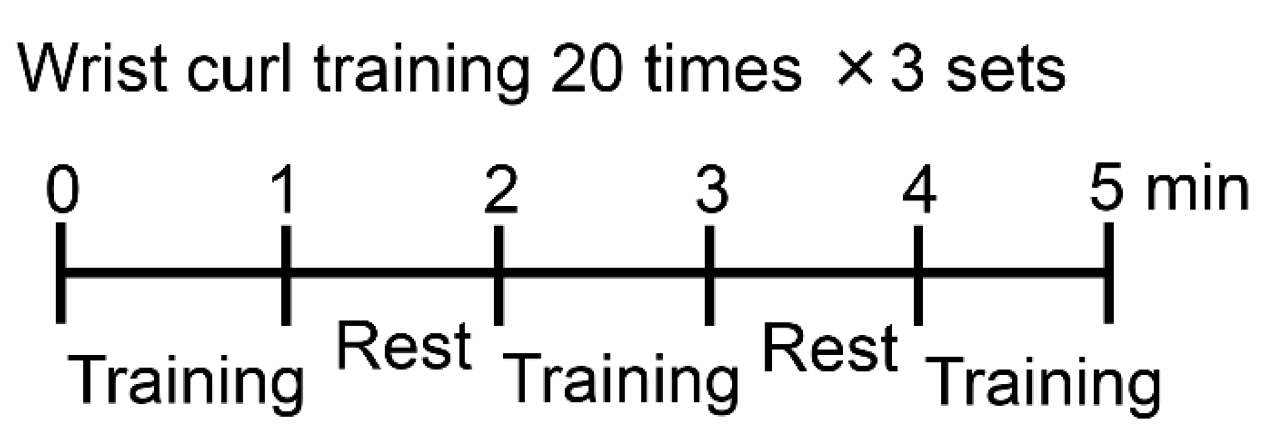


**Experimental time protocol of wrist curl training**

**fNIRS**

Functional near-infrared spectroscopy (fNIRS) uses near-infrared light from 700 nm to 900 nm. This is a non-invasive method for estimating the hemoglobin (Hb) concentration in blood from the difference of oxygenated hemoglobin (oxyHb) and deoxygenated hemoglobin (deoxyHb) in the near-infrared absorption spectrum as blood oxygen dynamics There are several different measurement methods, and the most commonly used is the continuous wave (CW) method, and the attenuation of near-infrared light intensity in the tissue is based on Lambert-Beer law. The concentration changes of oxyHb, deoxyHb, and total hemoglobin (totalHb), which is the sum of both, are obtained using multiple wavelengths, but the obtained signals are the products of the concentration changes and the optical path lengths that passed before the irradiation light was detected in the living body. When the intensity of the incident light is *I*_in_ and the intensity of the detected light is *I*_out_, the following equation holds:


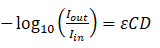
 (2)

where *ε* is the extinction coefficient, *C* is the hemoglobin concentration in the tissue, and *D* is the optical path length. In NIRS, because of the influence of disturbances due to external light and blood flow, the following modified Lambert-Beer law, which is an extension of Lambert-Beer law and is adapted to random scattering media. When *S* is attenuated by light scattering, the following equation holds:


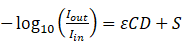
 (3)

where, assuming that the hemoglobin concentration changes to *C* + Δ*C* and the intensity of the detected light changes to *I*_in_ + Δ*I*_out_, the following equation holds:


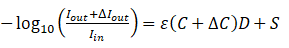
 (4)

where, assuming that the change in the intensity of the detected light is Δ*A*, the following equation holds from the above two equations:


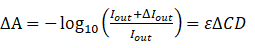
 (5)

There are two types of hemoglobin in the body, oxygenated hemoglobin (oxyHb) and deoxygenated hemoglobin (deoxyHb). Assuming that the absorption coefficients are Δ*ε*_oxy_ and Δ*ε*_deoxy_, and the concentration changes are Δ*C*_oxy_ and Δ*C*_deoxy_, the following equation holds:


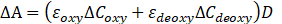
 (6)

In the above equation, in order to obtain the concentration changes, Δ*C*_oxy_ and Δ*C*_deoxy_ of oxyHb and deoxyHb, it is necessary to perform measurement with at least two types of light sources having different wavelengths.

The fNIRS monitoring device used in this experiment is Pocket NIRS Duo (DynaSense, Hamamatsu, Japan) shown in the figure below.


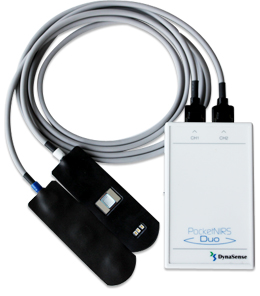


**fNIRS monitoring device**

Since this device uses LED light sources with three wavelengths, 735 nm, 810 nm, and 850 nm, the principle is that values can be calculated by solving the following simultaneous equations with *λ*_1_ = 735 nm, *λ*_2_ = 810 nm, and *λ*_3_ = 850 nm:


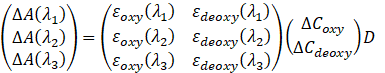
 (7)

As shown in the figure below, the middle part of flexor carpi radialis muscles and the proximal part of flexor carpi radialis muscles of the left upper arm were selected as CH-1 and CH-2, respectively, and the sampling time was set to 1 Hz as the monitoring points by the NIRS monitoring device. MF exposure was applied to the back of the forearm for 15 min immediately after the end of the muscle loading exercise. NIRS measurements were performed by placing the back of the forearm on an MF exposure device for 25 min (15 min of exposure and 10 min after exposure) with the hand and arm as stationary as possible. In this study, the hemoglobin oxygenation index (HOI) values (ΔoxyHb – ΔdeoxyHb concentration) were calculated from the concentrations of ΔoxyHb, ΔdeoxyHb, and ΔtotalHb obtained as measured values, and the comparisons were made between MF and sham.


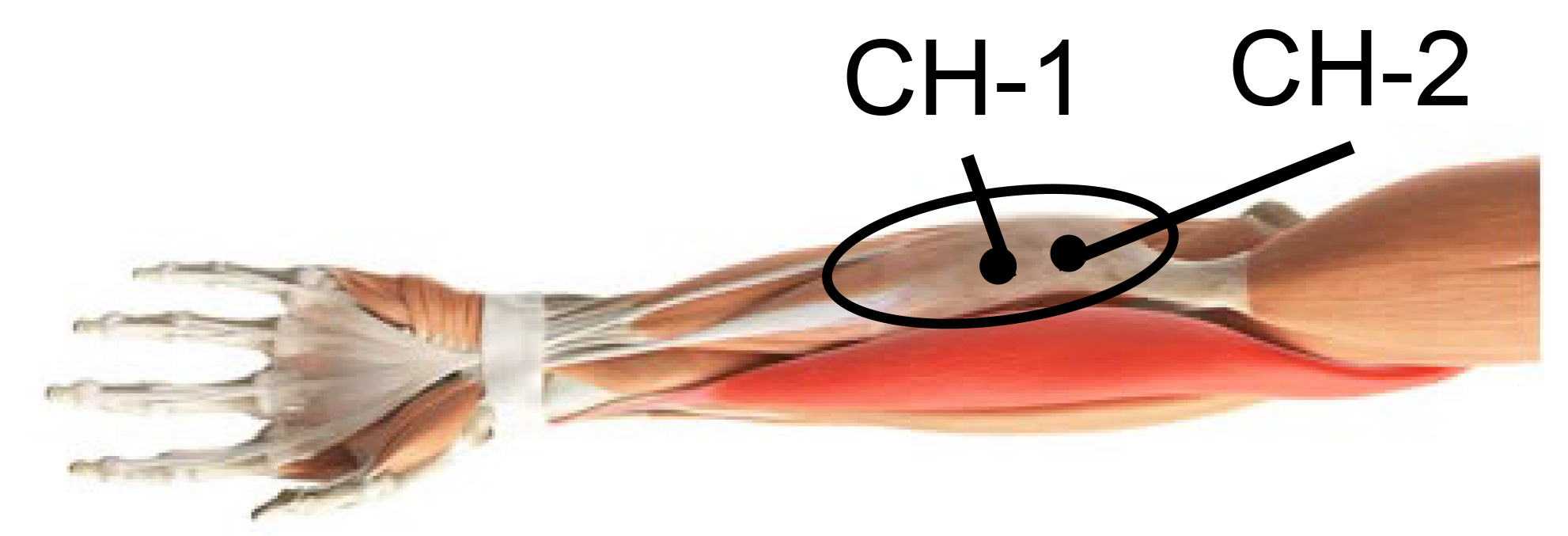


Monitoring points


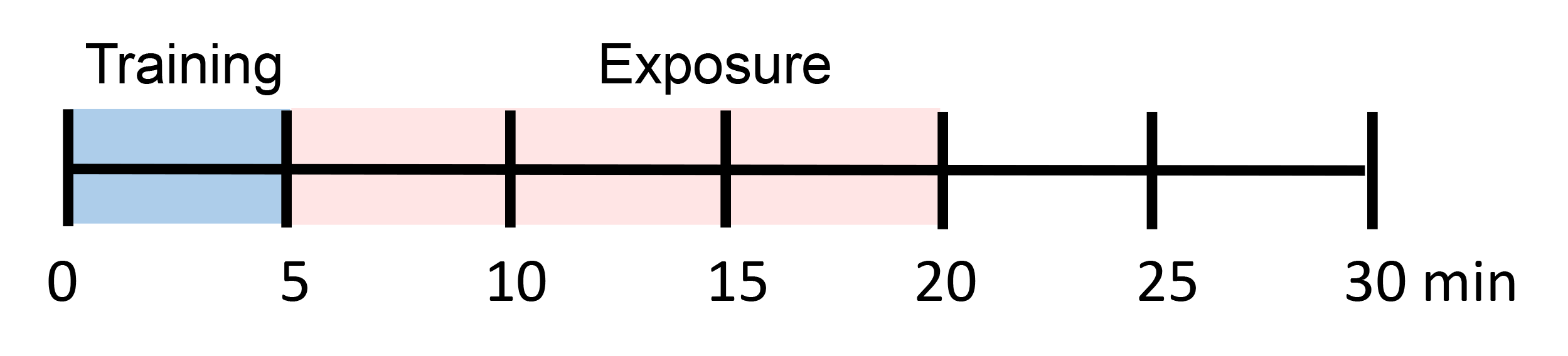


**Experimental time protocol**


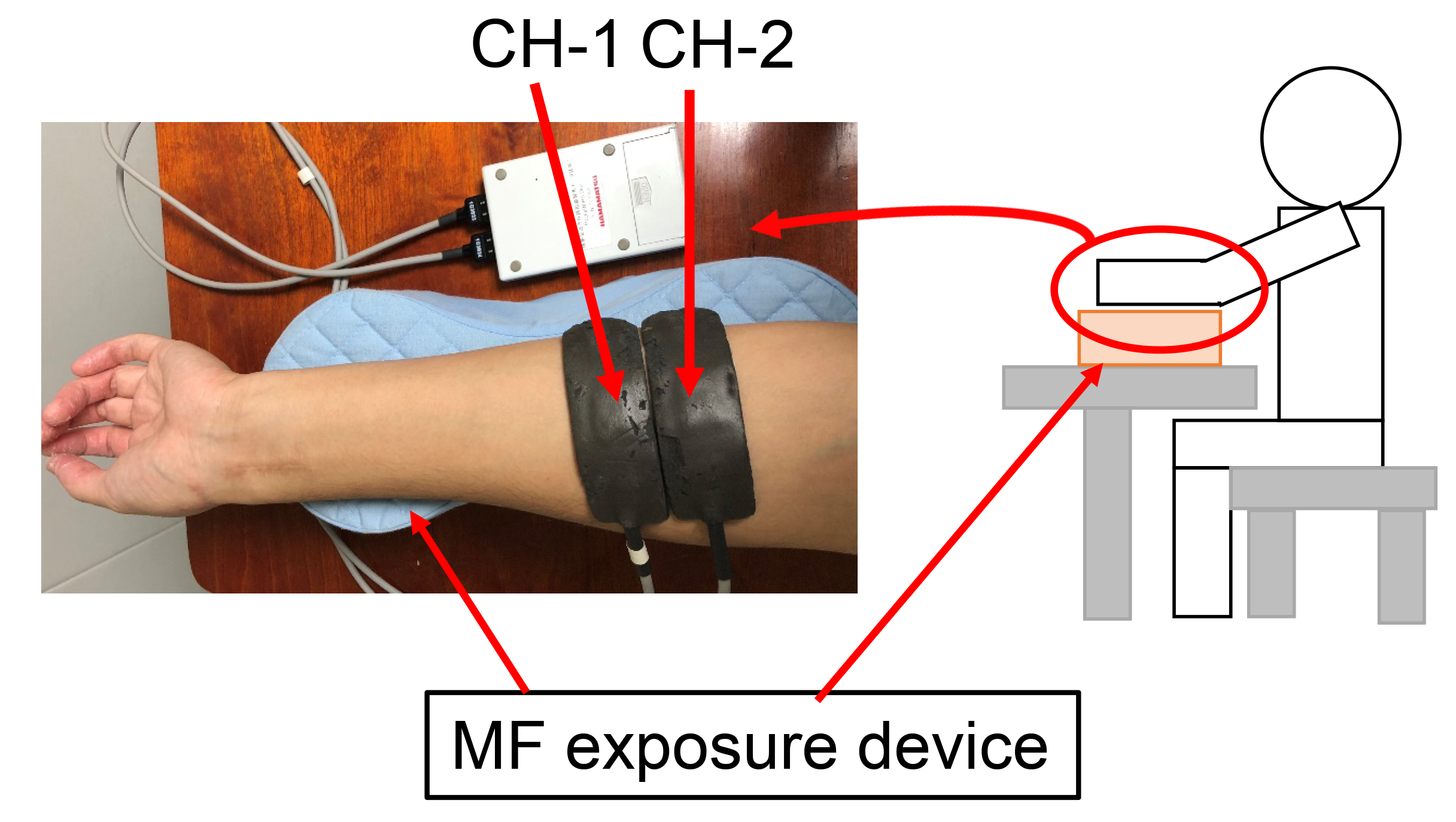


**ECG monitoring and forearm exposure**

**ECG**

The ECG waveform is composed of P wave, QRS wave, T wave, and U wave. The P wave indicates the excitement of the right and left atrium, the QRS complex indicates the excitement of both ventricles, the T wave indicates the process of extinction of ventricular excitement, and the U wave indicates dilation of the ventricles.


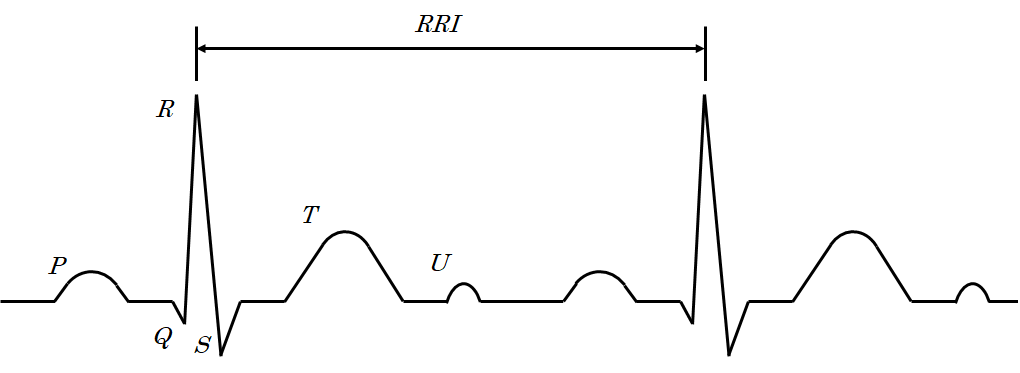


**Schematic diagram of ECG waveform**

It is possible to know the cardiac autonomic nerve activity to some extent by analyzing the RR interval (RRI) alone, which is the time interval at which the R wave is generated, even in the heartbeat generating the above-mentioned P-Q-R-S-T-U waves as one cycle. In this study, the ECG signal was extracted with noise removed from the ECG waveform captured at 250 sample/s for analysis. Furthermore, the ECG signal was analyzed by Fast Fourier Transform (FFT), the frequency and power were extracted, and after that, the frequency range of 0.05–0.15 Hz was defined as the low frequency component (Low Frequency: LF), and the frequency range of 0.15 to 0.40 Hz. was defined as the high frequency component (High Frequency: HF).

In general, the LF component corresponds to the amount of sympathetic nerve activity activated in the active or tensive state, and the HF component corresponds to the amount of parasympathetic nerve activity activated in the resting or relaxed state. However, since it is known that the LF component also increases when the parasympathetic nerve is activated, it was not used as the evaluation index this time. This is because the LF component fluctuates depending on the influence of both the parasympathetic nerve and the sympathetic nerve. Instead, we calculated LF/HF, which is considered to be an index of sympathetic nerves. Thus, in this study, we decided to use HF as an evaluation index for parasympathetic nerves and LF/HF as an evaluation index for sympathetic nerves.

A multi-channel telemeter system (WEB-1000, Nihon Kohden Co., Ltd., Tokyo, Japan) was used as the ECG monitoring device, and as shown in the figure below, electrodes were attached to the chest and ECG was monitored non-invasively for 25 min. The ECG was measured by the same method as in the neck exposure experiment, and the MF exposure was on the back side of the neck. The ECG monitoring for 20 min including MF or sham exposure for 15 min was performed with the subject in a seated position.


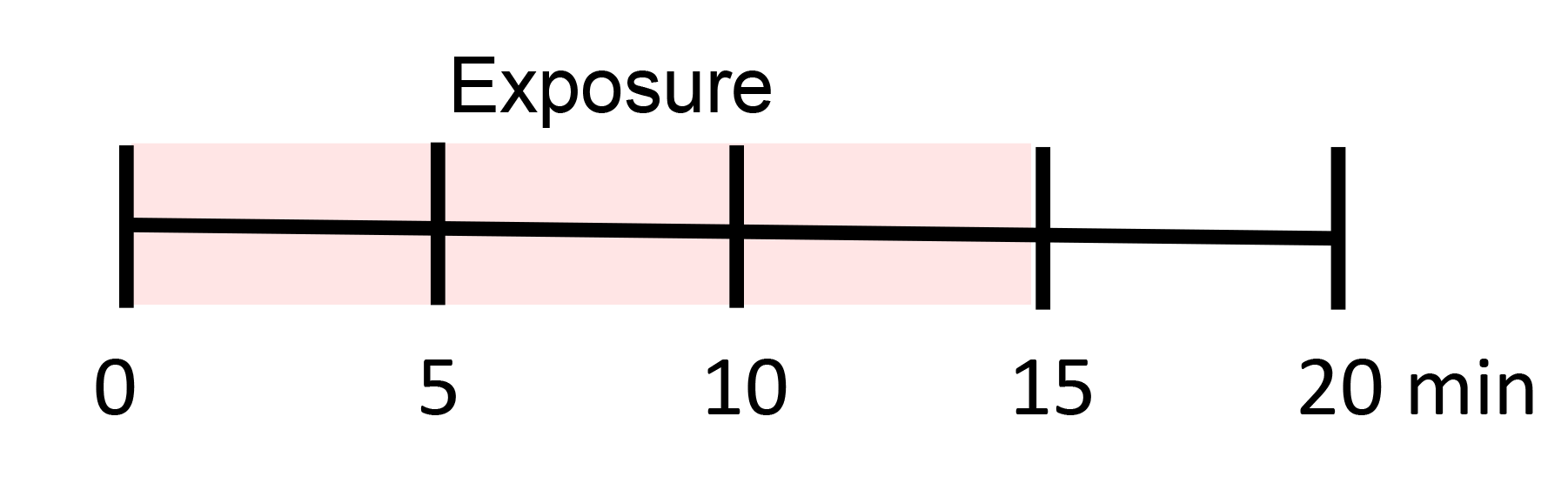


**Experimental time protocol**


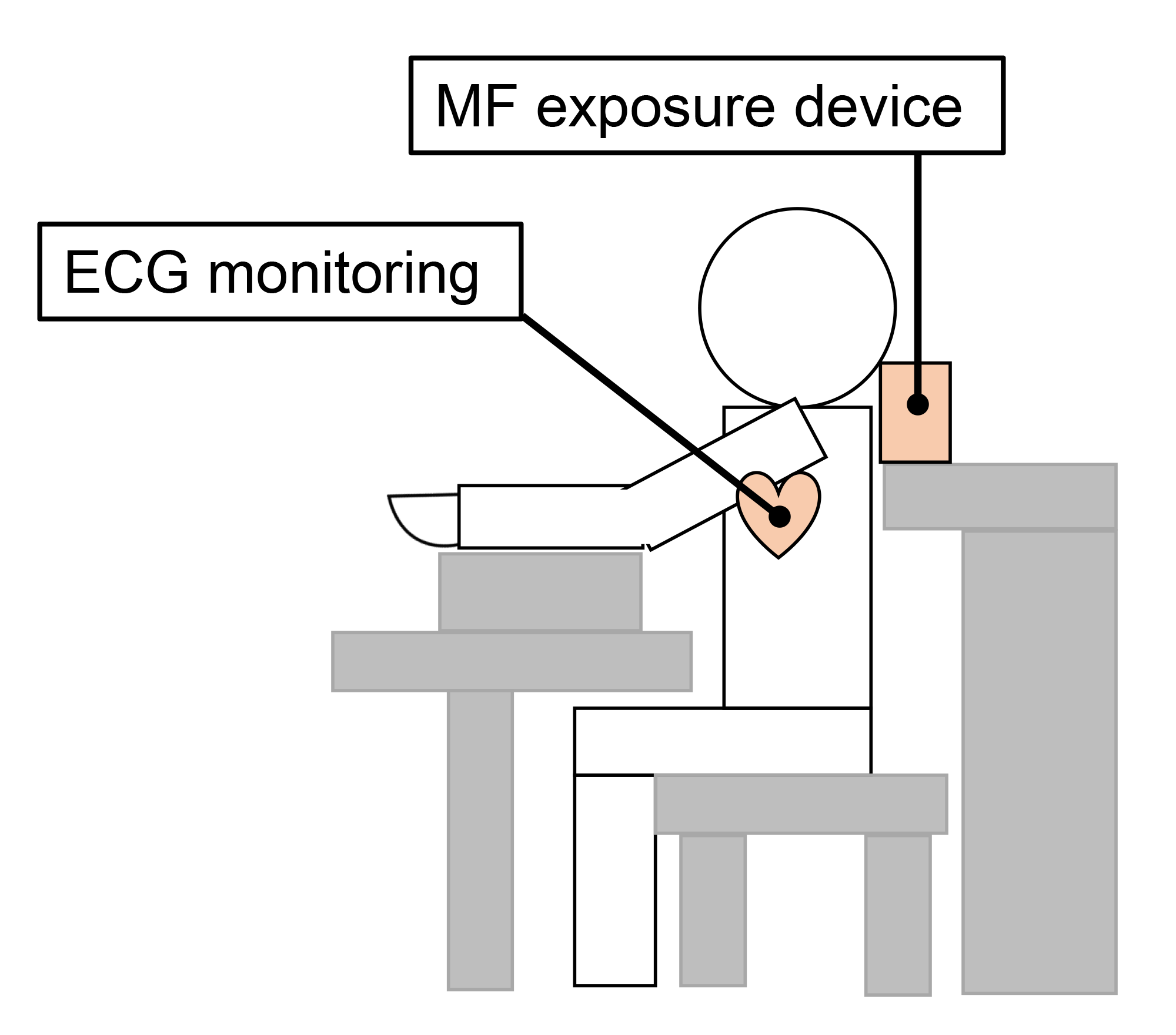


**ECG monitoring and neck exposure**

**FMD**

Flow-mediated dilation (FMD) is often utilized to noninvasively assess the vascular function, sometimes referred to as vascular endothelial function. In the FMD test, nitric oxide (NO), which is a vasodilator, is released from the vascular endothelium due to shear stress caused by increased blood flow after occluding the blood flow with a pressure-​controlled fast-inflating cuff. The vascular endothelial function is investigated by measuring the amount of vasodilation that occurs in response to these stimuli and calculating it as the ratio of changes in blood vessel diameter. When the vascular endothelial function is reduced, NO production is reduced and consequently, FMD levels are reduced. The FMD value was obtained by the following equation:


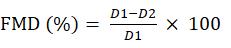
 (8)

where *D*1 = the basal diameter and *D*2 = the maximum diameter reached after cuff release (decompression). Thus, FMD values were automatically calculated as the percentage change in peak vessel diameter from the baseline value.

The FMD monitoring device used in this study is UNEXEF 18VG (UNEX Co., Ltd., Nagoya, Japan) in the figure below. The characteristics of this device are that it is equipped with a special H-shaped probe that can extract a long-axis image and two short-axis images at the same time. Therefore, it is easy to adjust the probe to a point where the blood stream and the ultrasonic beam are orthogonal. Furthermore, even if the blood vessel position shifts, it is possible to know which direction it is moving, and it is also possible to automatically track the blood vessels without losing track of their position.


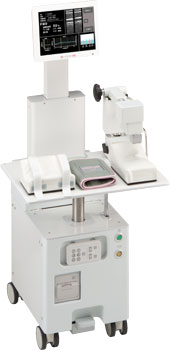


**FMD monitoring device**


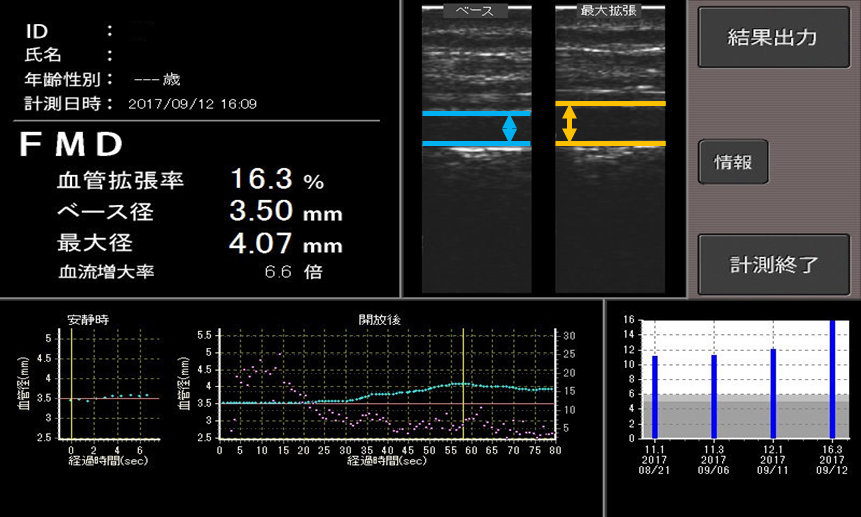


**The results of FMD monitoring**

Before the FMD monitoring, the subject placed the left upper arm on the MF exposure device in a supine position on the bed and attached a cuff to the left forearm. The subject was rested for 5 min or longer, and the dorsal side of the upper arm was exposed to an MF for 30 min. The FMD monitoring of the brachial artery was performed before and after the exposure. The reason why the MF exposure was set to 30 min is that since in the brachial artery FMD monitoring, the cuff pressure is applied to the FMD monitoring points, it is necessary to leave a monitoring interval of 30 min or more to eliminate the influence of the cuff pressure loading. The FMD monitoring points in the left brachial artery by the ultrasonic probe were set to the points of 50–80 mm from the elbow to the proximal side according to the length of the subject’s arm, and the MF exposure was adjusted to be directly under the blood vessel. The FMD monitoring after adjusting the monitoring points is fully automatic, and it takes about 7–10 min to obtain one test result. That is, after measuring the baseline artery diameter at rest, blood occlusion is performed for 5 min using an occlusion cuff, the artery diameter was continuously monitored for 2 min after cuff release, and the FMD value was calculated as the change in diameter from baseline to the maximum diameter reached after cuff release as shown in equation (8). It has been reported that there are two types of FMD monitoring, forearm artery occlusion, and brachial artery occlusion. In this experiment, forearm artery occlusion, which is highly NO-dependent, was used for FMD monitoring.


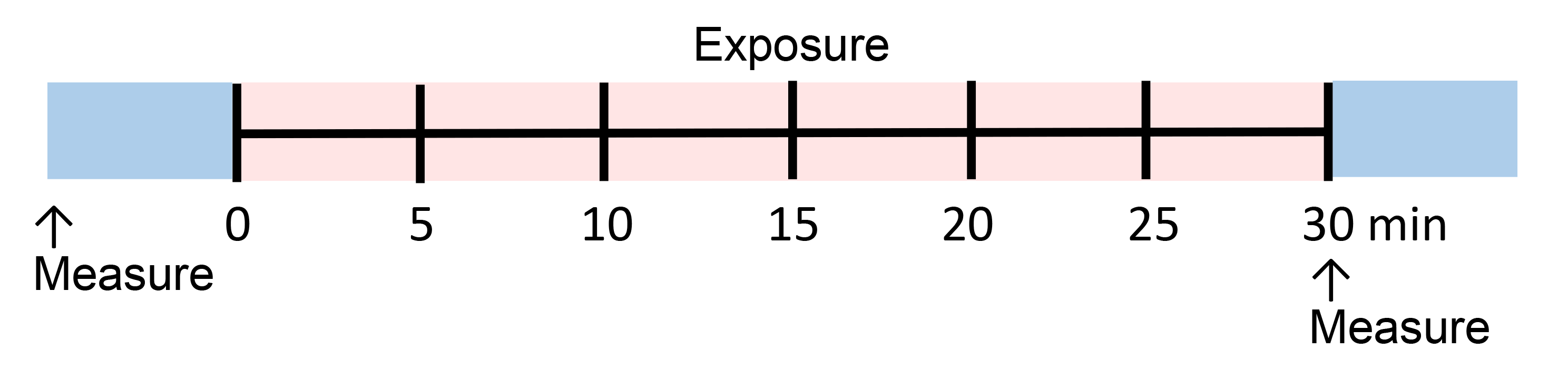


**Experimental time protocol**


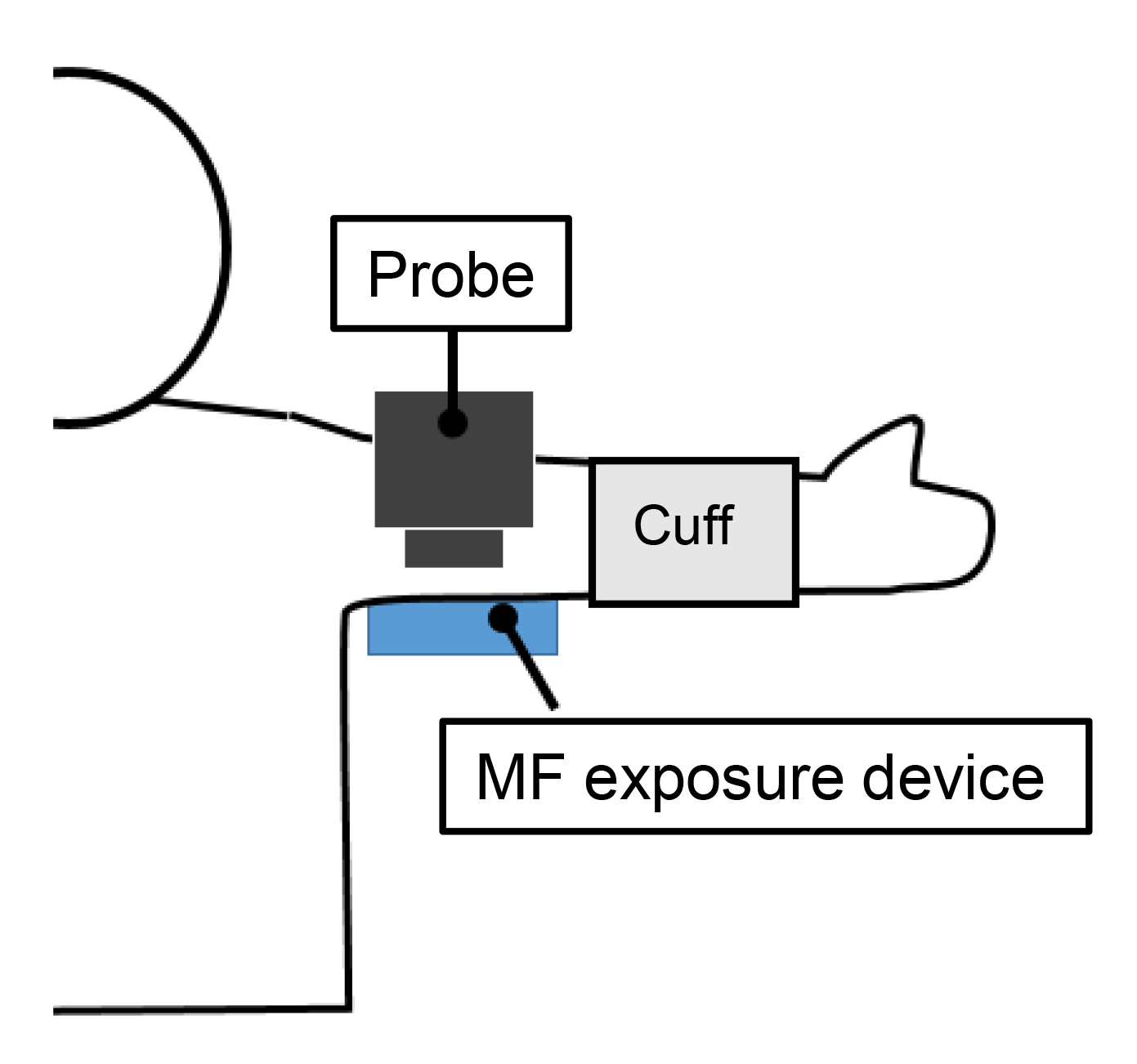


**FMD monitoring and upper arm exposure**
