# Supplementary material for: A 50 Hz magnetic field affects hemodynamics, ECG and vascular endothelial function in healthy adults: A pilot randomized controlled trial
Source: PLoS One. 2021 Aug 5;16(8):e0255242. doi: 10.1371/journal.pone.0255242 (PMC8341886; doi:10.1371/journal.pone.0255242)
Supplement: S2 File — (DOC) [file pone.0255242.s002.doc]

（様式１）

受付番号

　　　　　　　　　　　　　　　　　　　　　　　　　　　平成 29 年 5 月 8 日

埼玉大学倫理審査申請書

埼玉大学長　殿

　　研究責任者

　　所属　　大学院理工学研究科

　　職名　　教授

　　氏名　　綿貫 啓一　　　　　　　　　印

下記の研究計画について，国立大学法人埼玉大学におけるヒトを対象とする研究に関する倫理規則第１５条第１項の規定に基づき申請しますので，承認願います.

記

| １ 新規･変更の別 | ☑新規　・　☐変更 | | | | | | | |
| --- | --- | --- | --- | --- | --- | --- | --- | --- |
| ２ 課題名 | 交流磁場曝露が人体に及ぼす生理学的影響に関する評価 | | | | | | | |
| ３ 研究予定期間 | 審査結果通知書交付日　～　令和元年年10月31日 | | | | | | | |
| ４ 研究担当者  （他機関の共同研究者等がいる場合は記載） | ・埼玉大学教職員・学生等  ［所属・職名］　　　　　　　　　　　［氏名］  理工学研究科理工学研究科・教授　　　　　綿貫　啓一  兼任　先端産業国際ラボラトリー所長  理工学研究科理工学研究科　修士２年　　　藤村　明勝  理工学研究科理工学研究科　修士１年　　　近藤　司  先端産業国際ラボラトリー　非常勤研究員　岡野　英幸  ・共同研究者等  ［所属・職名］　　　　　　　　　　　［氏名］  なし | | | | | | | |
| ５ 研究実施場所  （他機関で研究が行われる場合は，他機関の倫理委員会の有無と審査結果等について記載） | ・埼玉大学構内  ［名称］研究機構棟5階　514実験室他  ・他機関の施設等  ［名称］  ［所在地］  ［倫理委員会］☐有／☐無  ［審査結果等］☐承認済／☐申請予定／☐申請不要（備考欄に理由を記載）  （備考：　　　　　　　　　　　　　　　　　　　）  なし | | | | | | | |
| ６ 研究の概要 | ［研究の目的，方法，期待される効果等］（簡潔に記載）  装着部位のこり及び血行の改善で厚生労働省より承認を受けている家庭用電気磁気治療器（交流磁気治療器）の臨床上の効果を最新の生体計測機器で評価し，ユーザ（潜在的なユーザを含む）の生理学的反応を客観的に評価する方法の開発を行う．生理学的反応の評価は，筋血流，末梢血流，近赤外線分光分析(NIRS)，血管内皮機能FMD検査，筋電図，筋硬度等の多角的な生理指標から特定する．  特に筋肉や血管の状態を可視化・数値化することにより，交流磁気治療に関する信頼性の高いエビデンスが得られ，さまざまな症状の改善や疲労回復につながる治療方法と治療器の開発に貢献し，より適切かつ効果的な処置情報（用法用量の適正化等に関する情報）を提供することができると考えられる． | | | | | | | |
|  | 研究の分類 | | ☐ヒトゲノム･遺伝子解析研究 | | | | | |
|  |  |  | ☐人を対象とする医学系研究 | | | | | |
|  |  |  | ☑人を対象とするその他研究 | | | | | |
|  | 個人情報の取扱い | | ☐無 | | ☑有　→　取扱う項目を下記に簡潔に記載 | | | |
|  |  |  | 氏名，年齢，性別，身長，体重，体温及び血圧 | | | | | |
|  | 人体から採取された試料等の使用 | | ☑無 | | 有　→　内容を下記に簡潔に記載 | | | |
|  |  |  | 尿（必要量[約5mL]を直ちに専用容器に移し，フリーザーに凍結保存し，随時，検査機関に検査委託[外注]する） | | | | | |
|  | 侵襲性 | | ☑無 | | ☐侵襲 　　　→ | | | 負担の種類  ☐身体的負担／☐精神的負担 |
|  |  |  |  |  | ☐軽微な侵襲 → | | |  |
|  | 介入の有無 | | ☐無 | | ☑有 | | | |
| ７ 研究対象者について | ［年齢層，性別，想定人数等］  20歳～90歳の健常者男女(アスリート含む)，約100人  包含基準:  理学療法を施行していない．  血管作用薬を服用していない．  体温，収縮期および拡張期血圧は正常範囲内．  実験参加同意書の署名あり．  ［募集方法］（研究対象者から除外する基準がある場合は明記すること）  20代の研究対象者（被験者）は埼玉大学の学生から募集し，30歳代～80歳代の研究対象者は一般から募集する．  除外基準  実験参加期間中に，上記包含基準を満たしていない場合．  ［謝礼･交通費等の有無］  一般から募集する30歳～90歳の研究対象者には実験時間に応じて謝礼を支払う． | | | | | | | |
|  | 未成年者 | | | ☑無 | | | ☐有　　→　　代諾者　☐有／☐無 | |
|  | 十分な判断力･意識 | | | ☑有 | | | ☐無　　→　　代諾者　☐有／☐無 | |
|  | ［代諾者の設定基準］（代諾者が必要な場合は記載） | | | | | | | |
|  | 疾病・障害 | | | ☑無 | | | ☐有　　→　　下記に配慮方法等を記載 | |
|  | ［疾病・障害を持つ研究対象者への配慮方法］ | | | | | | | |
| ８ 研究対象者への説明方法及び同意の取得方法  （研究参加の任意性等，説明･同意内容を具体的に記載） | 実験参加者（被験者）には事前に説明文書を用いて説明を行う．  以下について文書により同意を得る．  実験への参加は任意であり，気分や体調がすぐれない場合には実験の途中でも中止することができる．取得した実験データは統計的に処理され，個人が特定されることはない．実験の終了後であっても申し出により実験への参加を取りやめ，実験データを破棄することができる． | | | | | | | |
| ９ 実験等の実施手順  （箇条書きで具体的に記載） | １．被験者に交流磁気治療器を呈示する．  ２．被験者に対して無作為二重盲検法（参加者からも測定者からも不明の方法）により，交流磁気の曝露試験（曝露か非曝露かのどちらかの試験）を行う．その際，曝露か非曝露かの割り付けは，試験管理者（参加者，測定者以外の第三者）が行う．プラセボ効果や観察者バイアスの影響を防ぐためである．  ３．交流磁気曝露試験の実施およびその前中後（一定の時間間隔）あるいは連続的に生体データ(血流，NIRS，FMD，心電図，筋電図等)を取得する．  ４．上記試験は座位または仰臥位の姿勢で行う.  ５．オプションの試験として，筋疲労負荷試験（ダンベルによる重量負荷，トレッドミル等による運動負荷）と磁気治療を組み合わせた実験タスク前後等で，血流関連，筋電図等を計測する． | | | | | | | |
| １０ 成果の公表方法  （個人情報への配慮方法も記載） | 国内外の学術誌に投稿の他，国内外の学会で発表する．  公表時には個人を識別できる情報は一切記載せず，統計処理を行った後の要約値のみを記載する．  公表時には個人を識別できる情報は一切記載せず，統計処理を行った後の要約値のみを記載する． | | | | | | | |
| １１ 個人情報及び実験データ等の保護方法 | 実験データ等の匿名化 | | | ☐連結不可能匿名化して保管 | | | | |
|  |  |  |  | ☑連結可能匿名化し，対応表は別に保管 | | | | |
|  |  |  |  | ☐その他（　　　　　　　　　　） | | | | |
|  | 廃棄方法 | | | ☑学内規則等に基づき，一定期間保管した後，連結不可能匿名化して廃棄 | | | | |
|  |  |  |  | ☐その他（　　　　　　　　　　） | | | | |
|  | 他機関等への提供方法（該当する場合） | | | ☐対応表は提供せず，連結不可能匿名化の状態で提供 | | | | |
|  |  |  |  | ☐その他（　　　　　　　　　　） | | | | |
|  | ［具体的な保管･廃棄･提供方法や保存期間等］  まず，実験で回収したデータファイルは，インターネットから物理的に切断された記憶媒体を用いて，施錠環境にて保存する．次に，デジタルメディアに保存されたデータファイルについては，メディアを物理的に破壊し読取り不可能としたのちに廃棄する．紙媒体で記録されたデータについては，シュレッダーを用いて裁断した後に廃棄する． | | | | | | | |
| １２ 研究等によって生じうる不利益及び危険性，人権擁護の手法  （それぞれの事案への対策方法や，研究を中止する場合の基準等を具体的に記載） | 交流磁気治療器は，これまでにほとんど副作用や有害事象等の健康被害が報告されていないため，安全な治療器と考えられている．被験者は健常人であるため，本試験を実施するにあたって，「使用上の注意」や「禁忌・禁止事項」に該当する項目はない．  本研究の生体計測で用いる血管内皮機能FMD検査は，非侵襲の計測方法であるが，計測の際に検査時，仰臥位の状態で前腕にカフを巻いて5分間の駆血を行うため，駆血による痛みやしびれが発生する可能性がある（公開されている検査装置の製販業者による外部委託調査では，被験者の約2割にしびれの症状はあったが，痛みは全くなかったとの報告がある）．事前の体調を十分に確認したうえで実験を行うとともに，実験中も常に被験者を観察し，少しでも体調不良が認められる場合は実験を中止するなど危険性がないように努める．  実験データは高セキュリティの管理下に置き，データ分析の際には，デジタルデータの流出を防ぐためにセキュリティレベルの不安定なソフトのインストールは避け，ウイルスソフトの使用を充実させる．  実験者に対する問い合わせ，苦情の窓口を設け，苦情処理に対する速やかな対応体制を整え，全ての実験において，被験者の健康面，及び精神面に十分に配慮して実施する．また，各種実験器具の操作において，実験者は十分なトレーニングを行うことで人的ミスによる事故が起こらないように十分に注意する． | | | | | | | |
| １３ 添付資料 | ☑研究対象者への説明文書　☑同意書　☑その他（同意撤回書） | | | | | | | |
|  | ［添付しない場合の理由等］ | | | | | | | |
| １４ 主な研究資金 | ☐運営費　☐科研費（種別：　　　　　　　　　　代表者名：　　　　　　　）  ☑その他（　共同研究経費　） | | | | | | | |
| １５ その他実施上の留意点 |  | | | | | | | |
|  | | 部局長確認 | | | | 年　　月　　日 | | |

**臨床研究参加同意書**

実験協力者の方へ

年 月 日

**「交流磁場曝露が人体に及ぼす生理学的影響に関する評価」参画のお願い**

実験開始前にこの説明書をお読みいただき，ご協力いただける場合には，参加同意書に署名をお願いいたします．

研究の目的

現在，人体に交流磁場曝露を行うことによる有効性・安全性については，数多く報告されています．例えば，交流磁場によって疲労回復効果・皮膚温上昇効果がみられたという報告があります．しかしながら，人体でどのような生理学的な反応があるのかについての詳細な実施調査は行われていません．そこで本研究では，前腕の血流（尺骨動脈）のデジタルカラードップラー超音波診断検査，前腕屈筋部のヘモグロビン濃度変化の近赤外線分光分析（NIRS），心電図（ECG）の検査，上腕の血管内皮機能の血流依存性血管拡張反応（FMD）検査を行うことにより，交流磁場曝露による血流速度や組織酸素濃度，血管の状態に及ぼす影響を評価することを主な目的としています．

実験方法

血流速度

１．指定した椅子に着席し，左腕を磁場曝露装置の上に置きます．

２．5分間の安静後，実験者がプローブを前腕部に押し当て，血流速度を測定します．

３．安静後，5分間隔で血流速度を測定し，全6回の測定が完了すると1試行が終了となります．所要時間は1試行40分程度です．

NIRS

1. 指定した椅子に着席し，前腕屈筋部の二か所に２チャンネルのポケットNIRSのセンサーを装着します．
2. 左手(右利きの場合)で3 kgのダンベルを持ち，ダンベル・リスト・カール・トレーニングを5分間行います．

３．左腕を磁場曝露装置の上に25分間置いた姿勢を保ちます．その間の測定は連続的に自動的に行われます．

ECG

１．指定した椅子に着席し，頸部の後ろ側に磁場曝露装置を密着させます．

２．テレメトリー式の心電図計のプローブを胸部に貼付し，5分間安静にします．

３．その後も25分間同じ姿勢を保ちます．その間の測定は連続的に自動的に行われます．

FMD

１．指定したベッドに仰臥位で横たわり，左腕を磁場曝露装置の上に置きます．

２．駆血用のカフを左腕前腕部に装着し，プローブを上腕部に固定し，5分間の安静後，FMDを測定します．

３．FMDは1試行で２回測定します．1回の測定時間は約10分です．

４．FMDの２回目の測定は，1回目の測定後30分後に行います．仰臥位になって1試行が終了するまでの所要時間は約1時間です．

以上のいずれかの測定は1日に1試行とし，同様の測定は二日以上の間隔をあけた後に行います．

個人情報とデータの取扱い

本研究における個人を特定し得る個人情報の取得は，同意書の署名のみです．個人を特定し得ないデータや個人情報につきましても，研究目的以外には使用しません．データには番号付けを行うとともに匿名化しますので，専門学会，学術専門誌，学内研究会等を通じて研究発表する際も個人情報は守秘されます．データの保管には万全を期し外部へは漏洩しません．この保管データは，研究が終了してから5年後までに破棄します．

実験対象者の権利について

この研究に参加するか否かは自由意志で決定してください．また，一度同意した後においても同意を取り消すことができ，それによる不利益はありません．

匿名化番号を破棄するとともに，それまでに得られたデータや解析結果を破棄し，それ以降の研究には一切使用いたしません．但し，取り消し要求された時点で公表済みの解析結果がある場合は，このデータを破棄できませんのでご承知おきください．

実験に参加することによる利益と不利益

本研究に参加することによる費用の負担はありません．参加されなくても不利益を受けることは全くありません．在学生に対しては履修科目の成績評価とは関係ありません．先行学術研究例の範囲で，安全に留意の上で行います．不調が生じた場合には，実験を中止致しますので，実験の途中であっても速やかに実験者へお申し出下さい．

問い合わせ先について

本研究に参加している方の権利が守られていないと思われた場合や，担当者以外の意見や情報が欲しい場合は以下連絡先へご連絡ください．

(連絡先)

担当者：研究協力課 石川・齋藤

外線 048-858-9742，内線 3586，E-mail: kshinkou@gr.saitama-u.ac.jp

　研究の進捗や結果につきましては，実験者もしくは実験責任者へご確認下さい．

以上，何かご不明な点がありましたら遠慮なくお尋ねください．

本研究へのご理解とご協力に深く感謝いたします．

（実験者）

　埼玉大学大学院理工学研究科

藤村明勝，近藤司

　埼玉大学先端産業国際ラボラトリー

　　岡野英幸

（責任者）

埼玉大学先端産業国際ラボラトリー

　　岡野英幸

**臨床研究参加同意書**

**「交流磁場曝露が人体に及ぼす生理学的影響に関する評価」**

≪説明を受けた項目≫

□ 研究の目的

□ 実験方法

□ 個人情報とデータの取扱い

□ 実験対象者の権利について

□ 実験に参加することによる利益と不利益

□ 問い合わせ，苦情等の連絡先

□ 研究代表者の氏名，所属，職名

私は，以上の説明を理解し，本研究に参加することに同意します．

年 　月 　日

所 属： ＿＿＿＿＿＿＿＿＿＿＿＿＿＿＿＿＿＿

氏 名： ＿＿＿＿＿＿＿＿＿＿＿＿＿＿＿＿＿＿

**実験プロトコール**

**無作為化**

被験者は全員右利きであったが，実験管理者によるコンピュータで生成した乱数表に従って，被験者2名ずつを一つのブロックとしてブロックランダム化した後，すべての被験者を磁場曝露（MF）（A）か疑似曝露（sham）（B）の2通りの条件に均等になるように分割し，MF（A）かsham（B）のどちらかの曝露を初めて体験する条件の割り付けをランダムに行なった．本研究のランダムクロスオーバー割り付けは，2処理，4期間(2 × 4)の場合であるので，ABABまたはBABAのパターンでのランダム化を試みた．

**盲検化**

上記のMFとshamへの割り付けは，実験参加者にも測定者にも分からないようにして行った．さらに磁場曝露装置の操作スイッチを操作する実験管理者が，実験参加者と測定者との接触をもたないようにすることにより，MFかshamかの曝露条件を実験参加者と測定者に対して盲検化した．磁場曝露装置は，実験参加者が延長ケーブルを使用してリモートでオン・オフの操作を行った．測定者は，磁場曝露装置の操作スイッチがオンかオフかの確認をすることはできなかった．実験参加者も，磁場曝露装置がいつオンかオフであるのかについても知ることはできなかった．

**統計解析**

すべての被験者について，下記測定項目の計測をMFとshamにおいて，それぞれ別々の日に各2試行ずつ実施し，得られたMFとshamの値を比較し，有意差検定を行った．因子としての「時間」と「条件」による双方向反復測定​ANOVAを用いて，その相互作用の影響について解析した．さらにMFとshamの群間の差異の事後分析は，スチューデントの*t*検定またはウィルコクソン順位和検定（条件間）で行われ，MFとshamの各群内の差異の分析は，対応のある*t*検定またはウィルコクソン符号付き順位検定（同一条件内）により行った．上記はすべて両側検定を用いた．どの検定を用いて解析するかは，シャピロ・ウィルクによる正規性検定で，サンプルの分布が正規分布か否かを判別した上で行った．上記すべての検定は，*p* <0.05で有意差ありとした．

**血流速度**

血流速度の測定は，超音波エコーを用いた超音波ドプラ法による収縮期最大血流速度（Peak Systolic Velocity：PSV）により評価した．PSVの測定結果を超音波エコー診断装置の測定画面に示す．超音波ドプラ法は，照射した超音波が血管内を移動する赤血球によって反射することで音の周波数が変化するドプラ効果を用いて赤血球の移動速度，つまり血流速度を求める方法である．ここで，超音波プローブから照射される超音波入射角*θ*および周波数*f*_0_，赤血球が反射する超音波周波数*f*_d_，生体内の音速*C*とすると式（1）のように血流速度*V*が求められる:


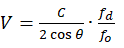
 (1)

ここで本研究では，超音波入射角*θ*をPSV測定に影響を及ぼさないとされている45～60度の範囲内に設定した．血管内を流れる血流は流体力学的に層流であることが判明している．そこで，下図の超音波エコー診断装置の測定画面に示すように，血流速度測定に必要なサンプルボリュームを，血管内径に対して約1/2～2/3程度に設定したが，その際，血管中央部でのサンプルボリュームの設定が難しいとされる大きく屈曲する血管は測定しないようにした．また，部位によって血管径が一定ではないことから，毎回の測定部位が同一部位でない場合，血流速度の測定誤差がさらに増大する可能性がある．そのため，毎回できる限り同一部位での測定が行えるように，初回の測定部位の皮膚表面に黒の油性マジックで目印をつけてから，2回目以降の繰り返し測定を行った．


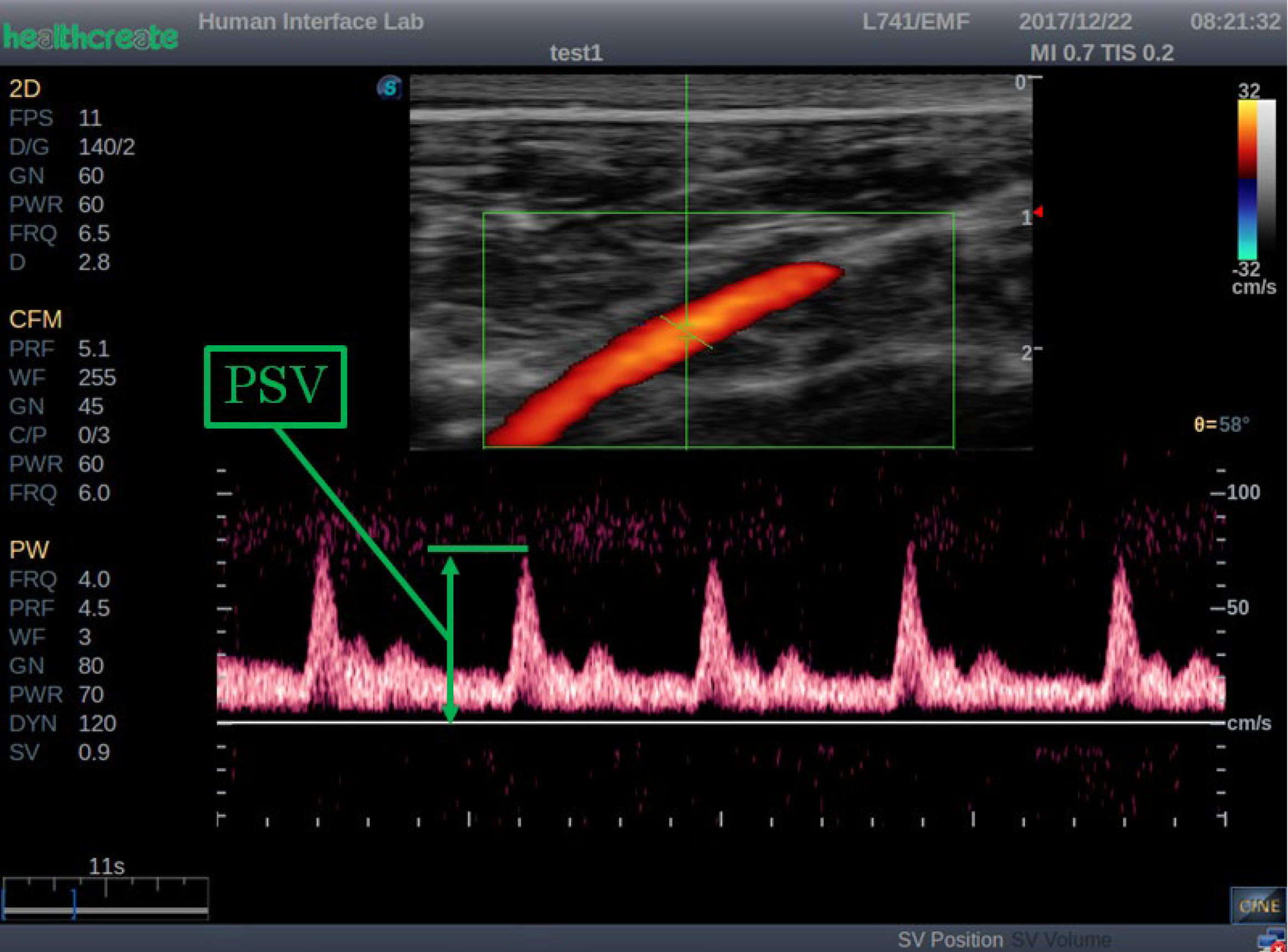


**PSVの測定結果**

PSV測定にはポータブル型デジタルカラードップラー付超音波システム（JS2，超音波プローブL741，Medicare株式会社，深圳，中国）を用いた．左前腕の腹側の皮膚表面に超音波プローブを接触させた状態で，腕尺骨動脈の血流速度を5分間隔で25分間測定を行った．被験者は左手のひらを上にし，左腕を120～140度曲げた状態を維持し，前腕部を磁場曝露装置の上に置き，5分間以上安静になった後，15分間のMFまたはsham曝露を行った．磁場曝露の部位は下図に示すように，前腕部，上腕部，あるいは頸部である．磁場曝露側は，前腕部と上腕部の場合が腕部背側，頸部の場合が頸部背側であった．いずれの場合にも前腕部は磁場曝露装置の上に静置した状態であった．上腕部と頸部の磁場曝露の際には，前腕部に使用した磁場曝露装置とは別の装置を使用し，前腕部への曝露は同時には行わなかった．

40度曲げた状態を維持し，前腕部を磁場曝露装置の上に置き，5分間以上安静になった後，15分間のMFまたはsham曝露を行った．磁場曝露の部位は下図に示すように，前腕部，上腕部，あるいは頸部である．磁場曝露側は，前腕部と上腕部の場合が腕部背側，頸部の場合が頸部背側であった．いずれの場合にも前腕部は磁場曝露装置の上に静置した状態であった．上腕部と頸部の磁場曝露の際には，前腕部に使用した磁場曝露装置とは別の装置を使用し，前腕部への曝露は同時には行わなかった．


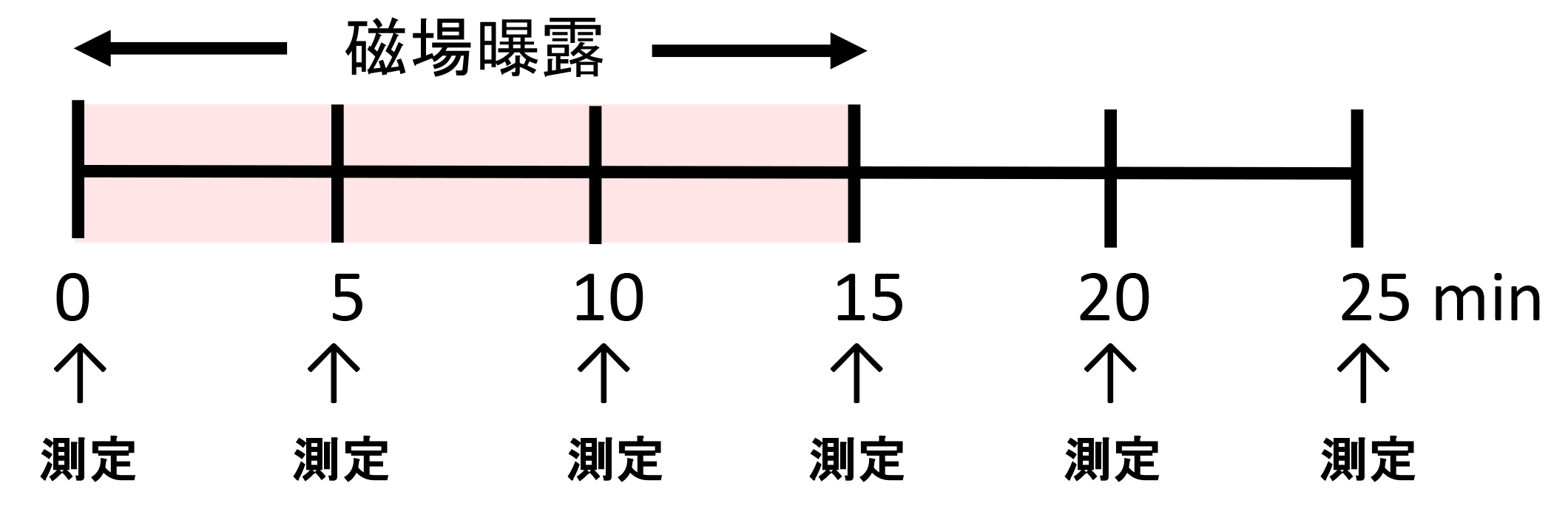


**実験タイムプロトコール**


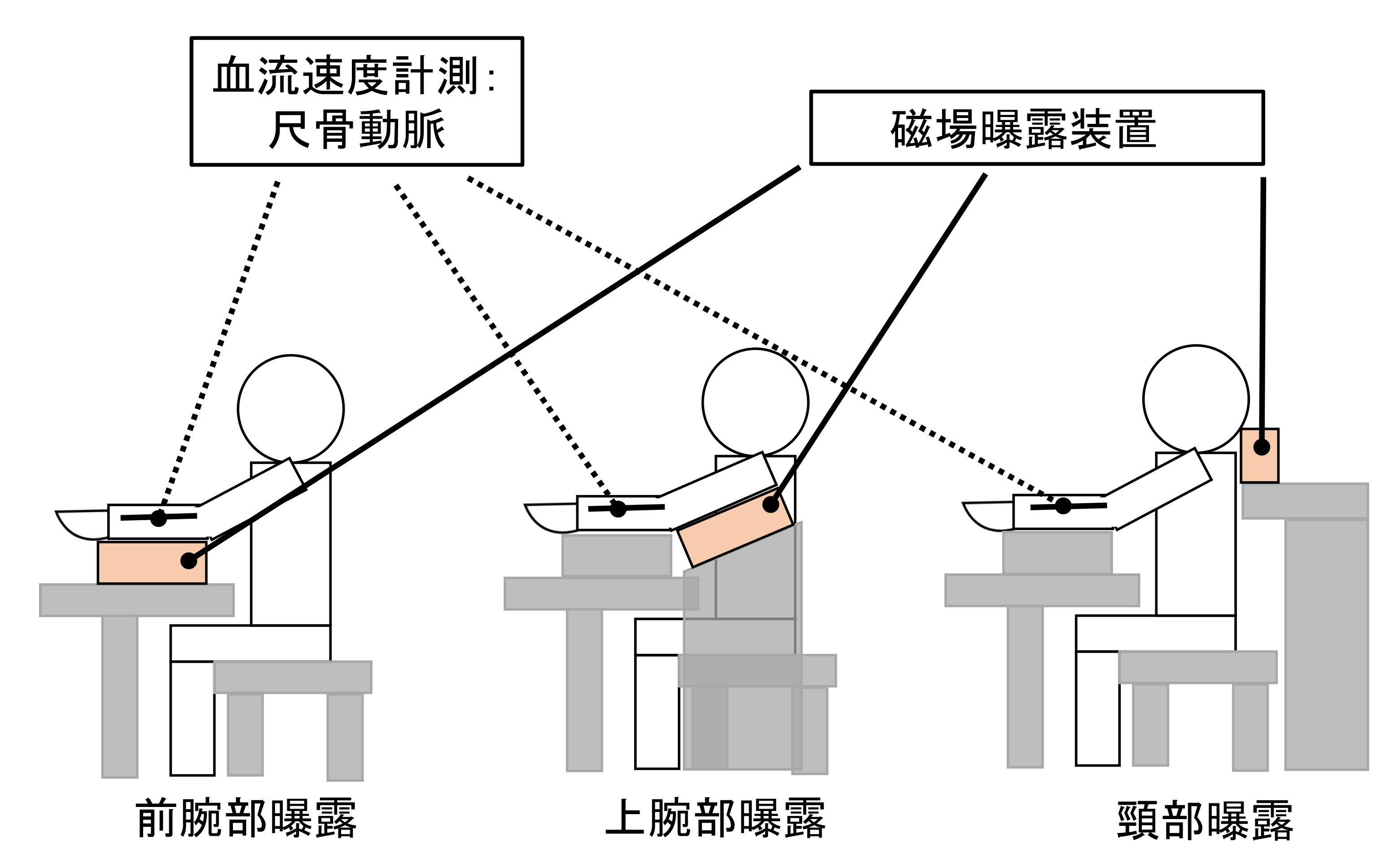


**磁場曝露部位と血流速度測定部位**

**血圧と心拍数の測定**

血圧と心拍数の測定は，前腕部曝露の実験と同様の方法により行った．下図に示すように上腕式デジタル血圧計（DSK-1051J，日本精密測器株式会社）を用いて10分間隔で磁場曝露前，曝露中，曝露後に測定を行った．


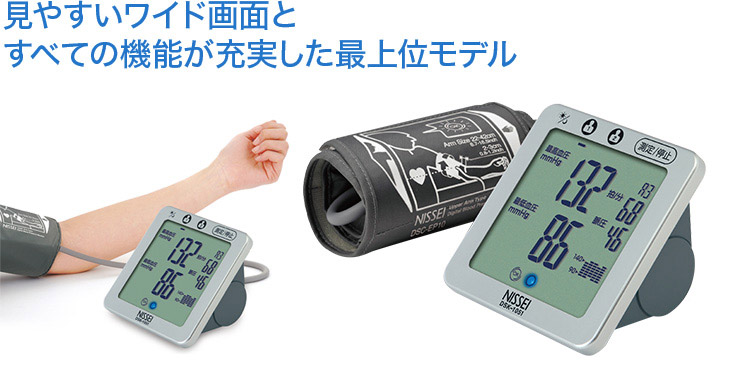


上腕式デジタル血圧計


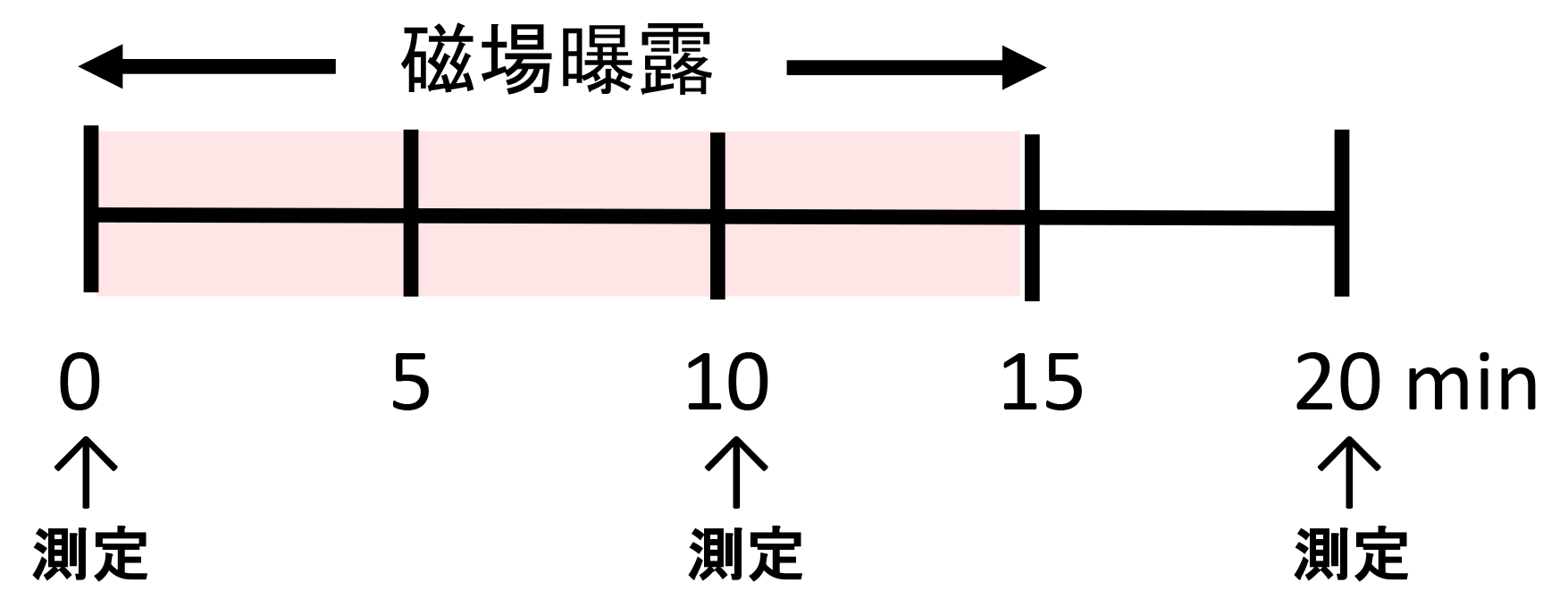


実験タイムプロトコル

**筋運動負荷方法の選択**

左前腕の手首屈筋における筋運動負荷（トレーニング）方法として最適な方法を選択するために，以下に示す三通りの方法による評価を行った．（１）前腕屈筋群のトレーニングには，ダンベルリストカールを行った．これは3 kgのダンベル（SINTEX Chrome array 3kg STW021，シンワ​エンタープライズ株式会社株式会社，大阪，日本）を用いて前腕の腹側を上に向けた状態で固定し手首の上げ下げする方法である．（２）前腕伸筋群のトレーニングには，リバースリストカールを行った．これは前腕の背側を上に向けた状態で固定し手首の上げ下げする方法である．（３）腕橈骨筋のトレーニングとしては，ハンマーカールを行った．これは肩から腕をまっすぐ下した状態で，ダンベルを手のひらが自分側に向くように持ち，肘を曲げ伸ばしする方法である．その結果，ダンベルリストカールを行った時が，他のトレーニングと比較してトレーニングの前後で手首屈筋における筋硬度が最も上昇したため，ダンベルリストカールトレーニングを選択した．

**リストカールトレーニング**

実験参加者は，下図に示すように，椅子に座った姿勢で5分間のトレーニングを行った．まず，実験参加者は，ダンベルを持ち，前腕が動かないようして，手のひらを上を向け，手首は自由に動かせる状態から，1秒間でダンベルを手関節背屈位まで上げ，2秒間でダンベルを手関節掌屈位の最大範囲まで下げた．１サイクルに3秒かかるため，1分間で20サイクルを行い1セットとし，その後1分間休息した．このようにしセット間に1分の休憩の間隔を設けて，5分間で20往復を3セット行った．


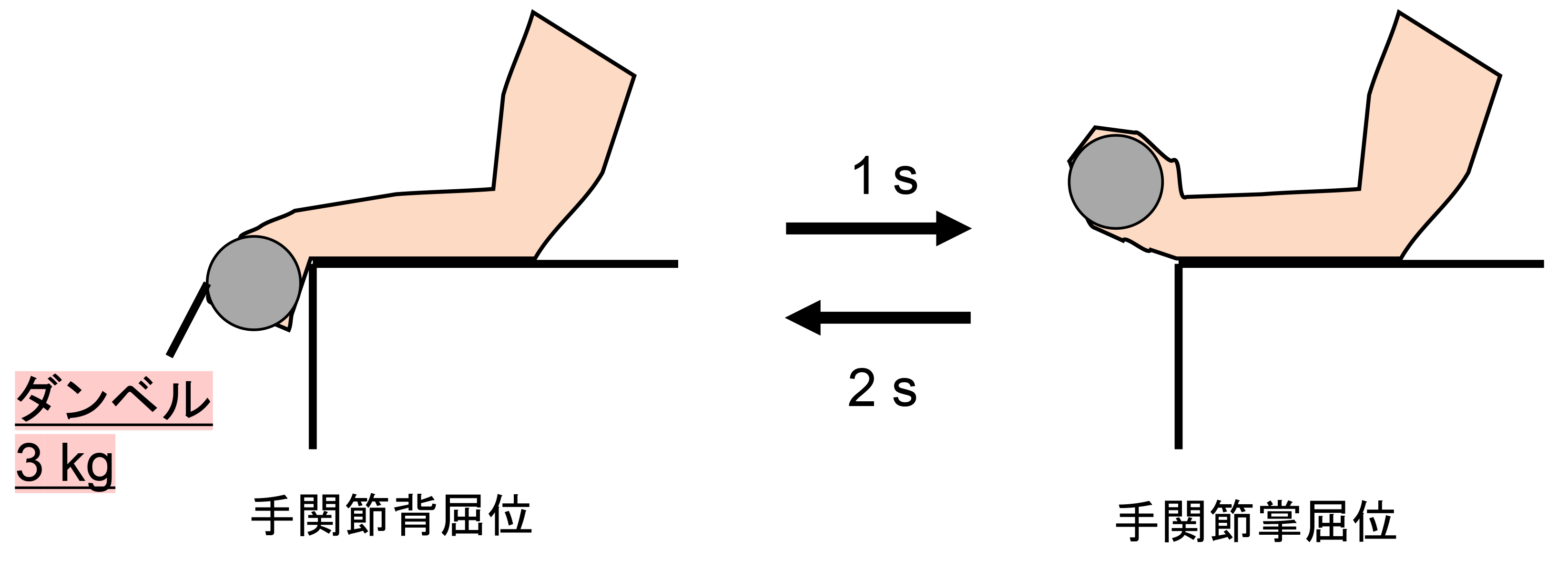


リストカールトレーニング


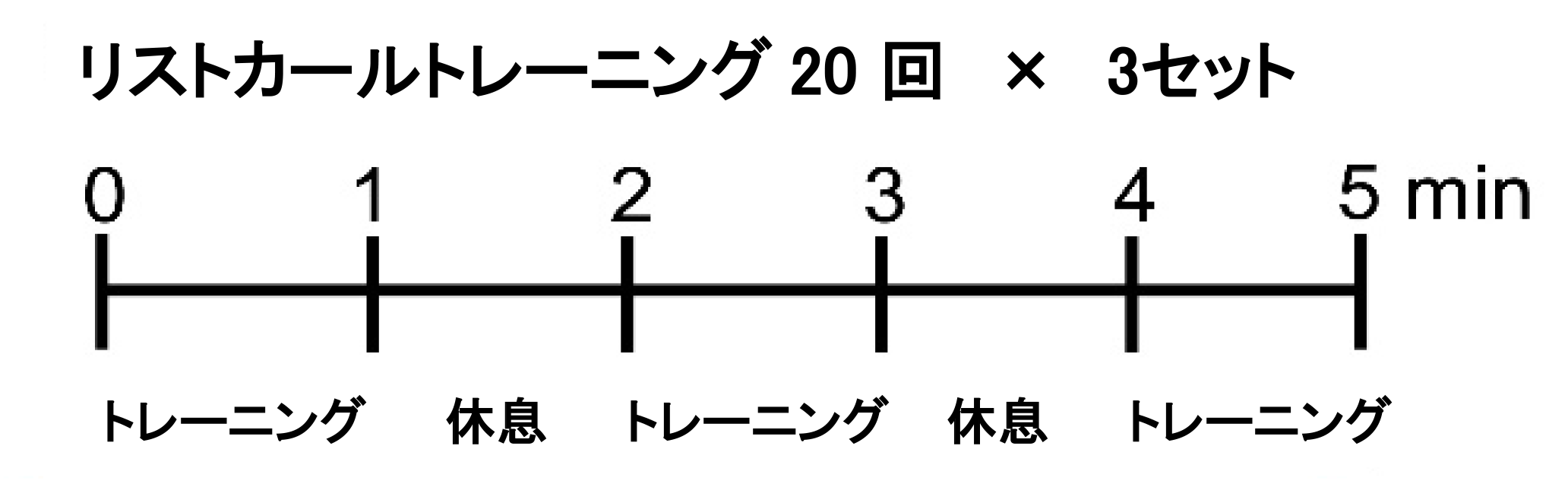


筋運動負荷プロトコル

**fNIRS**

機能的近赤外分光（fNIRS）法は，700 nm～900 nmの近赤外光を用いて，血中酸素動態として，酸素化ヘモグロビン（oxy-Hb）と脱酸素化ヘモグロビン（deoxy-Hb）の近赤外吸収スペクトルの違いから血中のヘモグロビン（Hb）の状態（濃度）を非侵襲で推定する手法である．その算出方法は，複数の異なる計測方法があるが，最も一般的に使用されているのが連続光法（CW計測）で，組織内の近赤外光強度の減衰はランベルト・ベールの法則（Lambert-Beer law）に基づいている．複数の波長を用いて酸素化ヘモグロビン，脱酸素化ヘモグロビン，両者の和である総ヘモグロビンの濃度変化を求めるが，得られる信号は濃度変化と光路長（照射光が検出されるまでに通った生体内における経路の長さ）の積となる．入射光の強度を*I*_in_と検出光の強度を*I*_out_とすると以下の式が成り立つ．


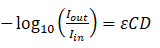
 (2)

ここでεは吸光係数，*C*は組織中のヘモグロビン濃度，*D*は光路長である．fNIRSでは，外部からの光や血流などによる外乱の影響があるため，ランベルト・ベールの法則を拡張した，散乱のある媒体に適応した以下の拡張ランベルト・ベール（modified Lambert-Beer law）の法則が適用される．*S*を光の散乱による減衰とすると以下の式が成り立つ:


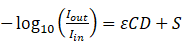
 (3)

ここでヘモグロビン濃度が*C*+Δ*C*検出光の強度が*I*_in_+Δ*I*_out_に変化したとすると，以下の式が成り立つ:


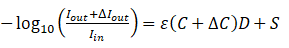
 (4)

ここで検出光の強度変化をΔAとすると，上の2式より以下の式が成り立つ:


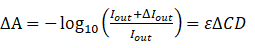
 (5)

生体内のヘモグロビンは酸素化ヘモグロビン(oxy-Hb)と脱酸素化ヘモグロビン(deoxy-Hb)の2種類であり，それぞれの吸光係数をΔ*ε*_oxy_，Δ*ε*_deoxy_，濃度の変化をΔ*C*_oxy_，Δ*C_deoxy_*とすると，以下の式が成り立つ:


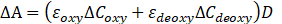
 (6)

上式において，oxy-Hbとdeoxy-Hbの濃度変化Δ*C*_oxy_，Δ*C_deoxy_*を求めるため，少なくとも2種類以上の波長が異なる光源により計測を行う必要がある．

本実験に用いたfNIRS計測装置は下図のPocket NIRS Duo（DynaSense株式会社, 浜松，日本）である．


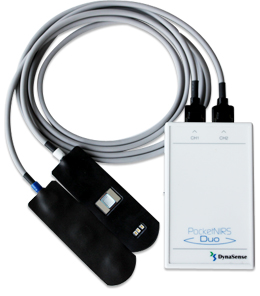


**fNIRS計測装置**

本装置では，735nm，810nm，850nmの3種類の波長のLED光源を用いているため，*λ*_１_ = 735nm，*λ*_2_ = 810nm，*λ*_3_ = 850nmとして，以下の連立方程式を解くことにより解を算出する原理となっている．


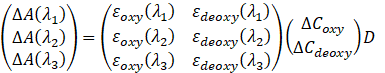
 (7)

fNIRS計測装置による測定部位として，下図に示すように，左腕上腕部の橈骨手根屈筋中央部，屈筋橈骨橈骨後部をそれぞれCH-1，CH-2として選定し，サンプリングタイムを1 Hzとした．

fNIRS計測は，前腕部曝露の実験と同様の方法により行った．磁場曝露は，筋運動負荷の終了直後から15分間，前腕背側に曝露した．fNIRS計測は，前腕背側を磁場曝露装置上に25分間（曝露15分および曝露後10分間）静置し，手と腕をできるだけ動かない状態にして行った．本研究では，測定値として得られたΔoxyHb，ΔdeoxyHb，およびΔtotalHbの濃度から，ヘモグロビン酸素化指数（HOI）値（ΔoxyHb – ΔdeoxyHb濃度）を計算し，MFとshamの間での比較を行った．


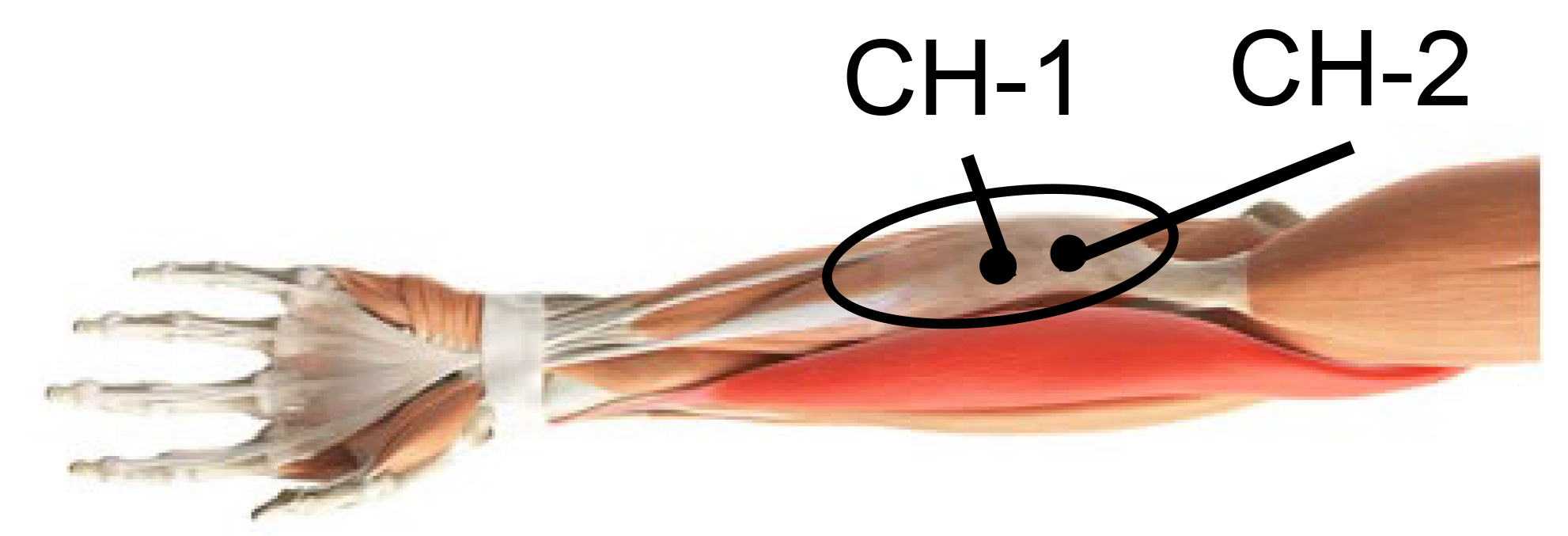


測定部位


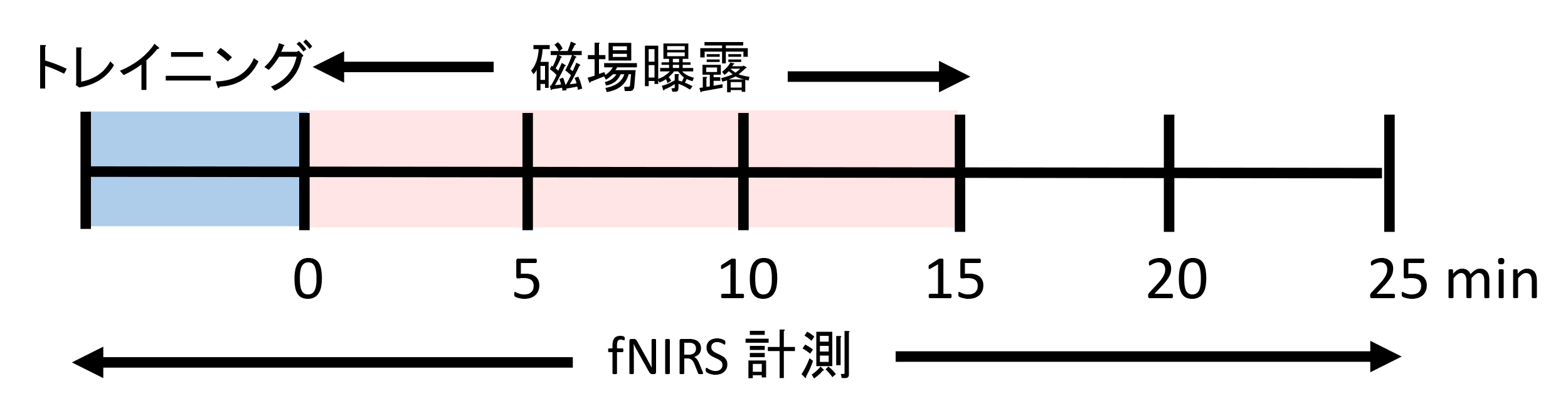


実験タイムプロトコル


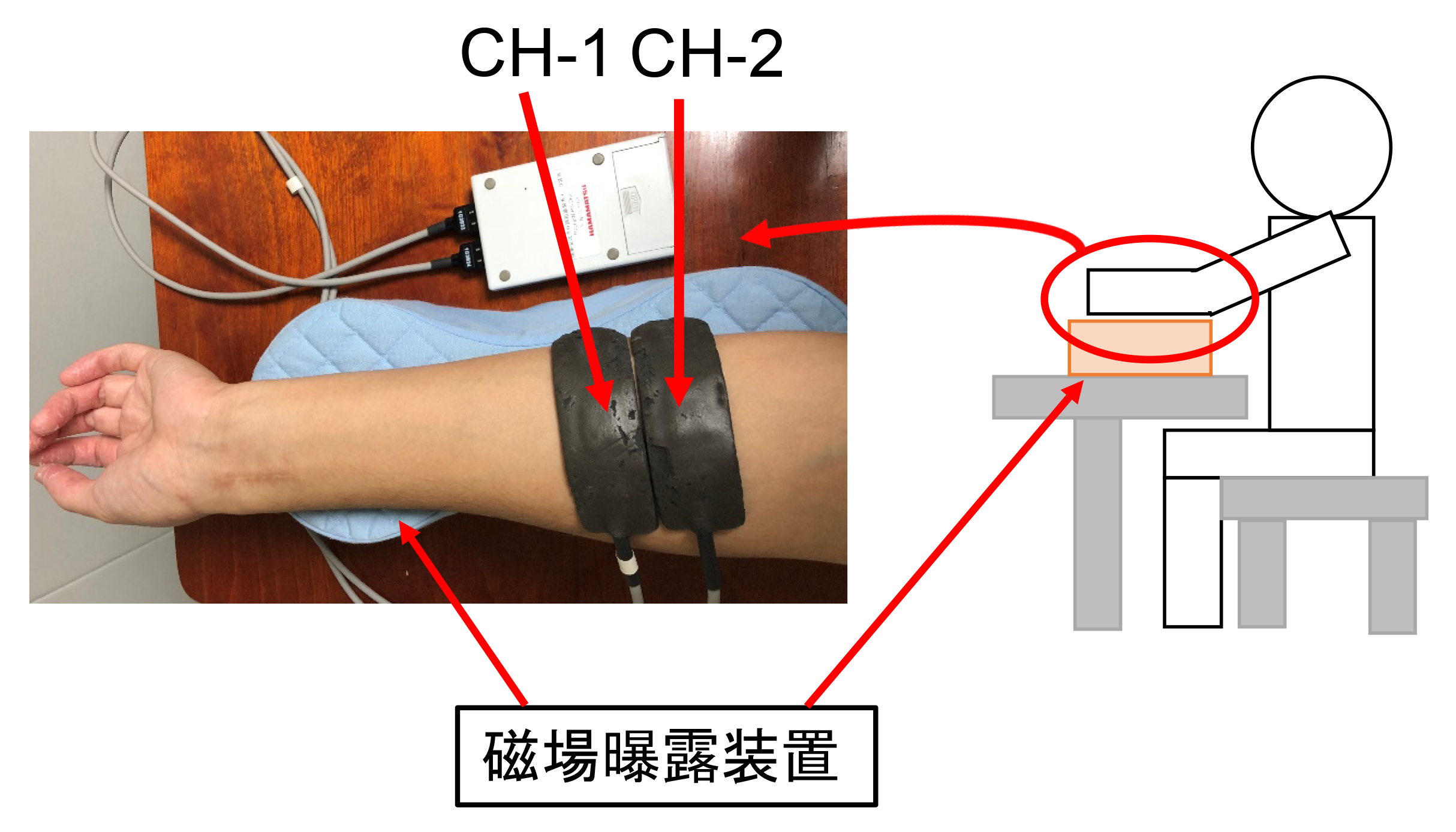


実験方法

**ECG**

ECG波形はP波，QRS波，T波，U波から構成されている．それぞれ，P波は右心房および左心房の興奮状態，QRS波は両心室の興奮状態，T波は心室の興奮が消退する過程，U波は心室の拡張を示している．


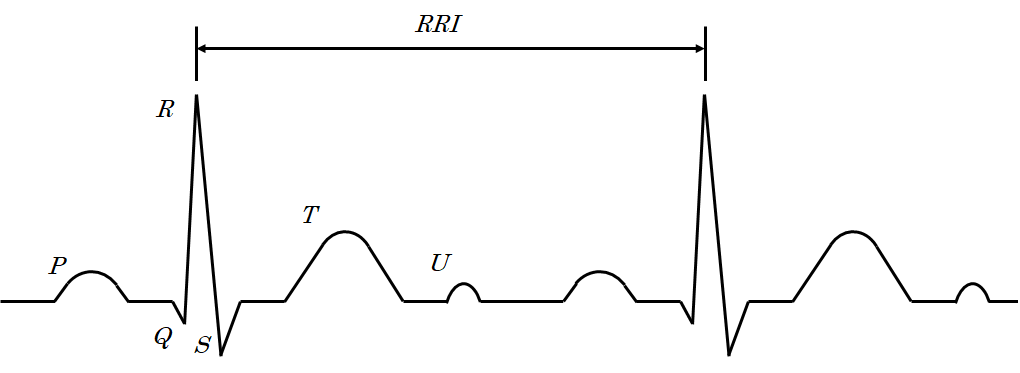


**ECG波形の概要図**

上記のP-Q-R-S-T-Uを1サイクルとした心拍のなかでも，R波が発生する時間間隔であるRR間隔（R-R interval：RRI）を解析することで，自律神経の活動をある程度知ることが可能である．本研究では解析を行うために，250 sample/sで取り込んだECG波形からノイズを除去したECG信号を抽出した．さらにそのECG信号を高速フーリエ変換（Fast Fourier Transform：FFT）により解析し，周波数とパワーを抽出し，0.05～0.15 Hzの周波数域を低周波数成分（Low Frequency：LF），0.15～0.40 Hzの周波数域を高周波数成分（High Frequency：HF）と定義した．

一般的にLF成分は活動状態や緊張状態において活性化する交感神経活動量，HF成分は休息時やリラックス状態に活性化する副交感神経活動量に相当するとされている．ただしLF成分は副交感神経が活性化した際にも上昇することが知られているため，今回の評価指標には用いなかった．LF成分は，副交感神経および交感神経どちらからの影響によっても変動するためである．その代わり，主として交感神経の指標であると考えられているLF/HFを算出した．このようにして本研究においては，HFを副交感神経の評価指標，LF/HFを交感神経の評価指標として用いることとした．

ECG測定装置には多チャンネルテレメータシステム（WEB-1000，日本光電工業株式会社，東京，日本）を用い，下図に示すように，胸部に電極を貼付し非侵襲的に25分間ECGを測定した．ECGの測定は，頸部曝露の実験と同様の方法により行い，磁場曝露は頸部背側に曝露した．15分間のMFまたはsham曝露を含む20分間のECG測定は，被験者が座位の姿勢で行われた．


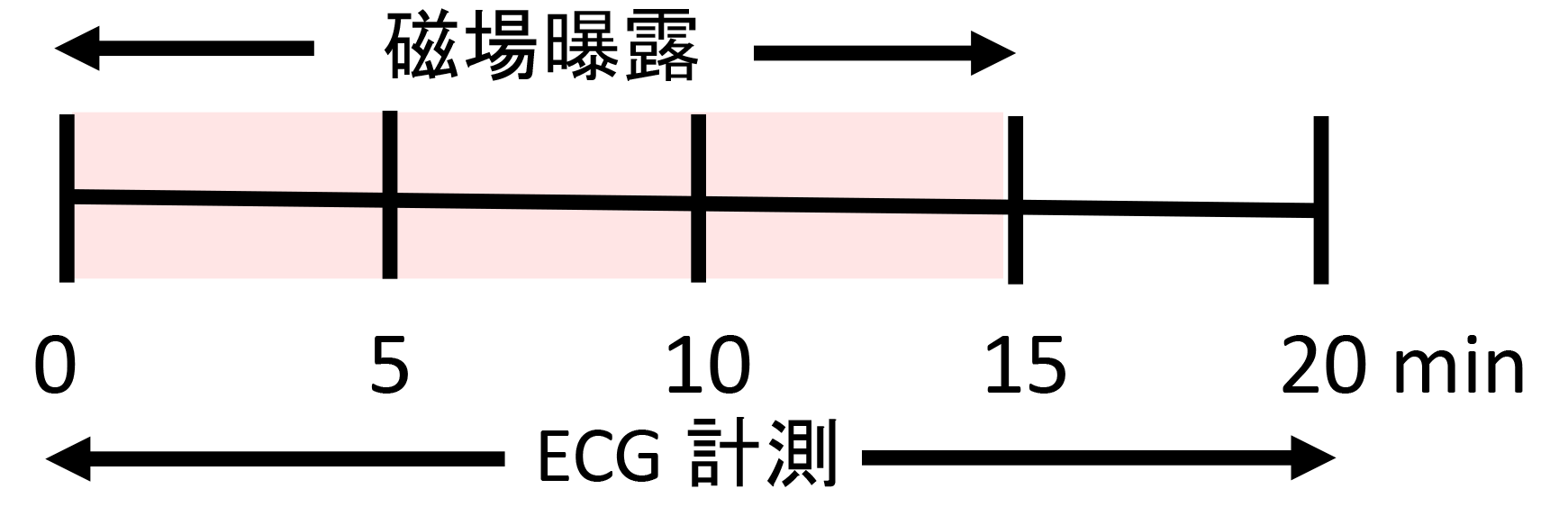


**実験タイムプロトコール**


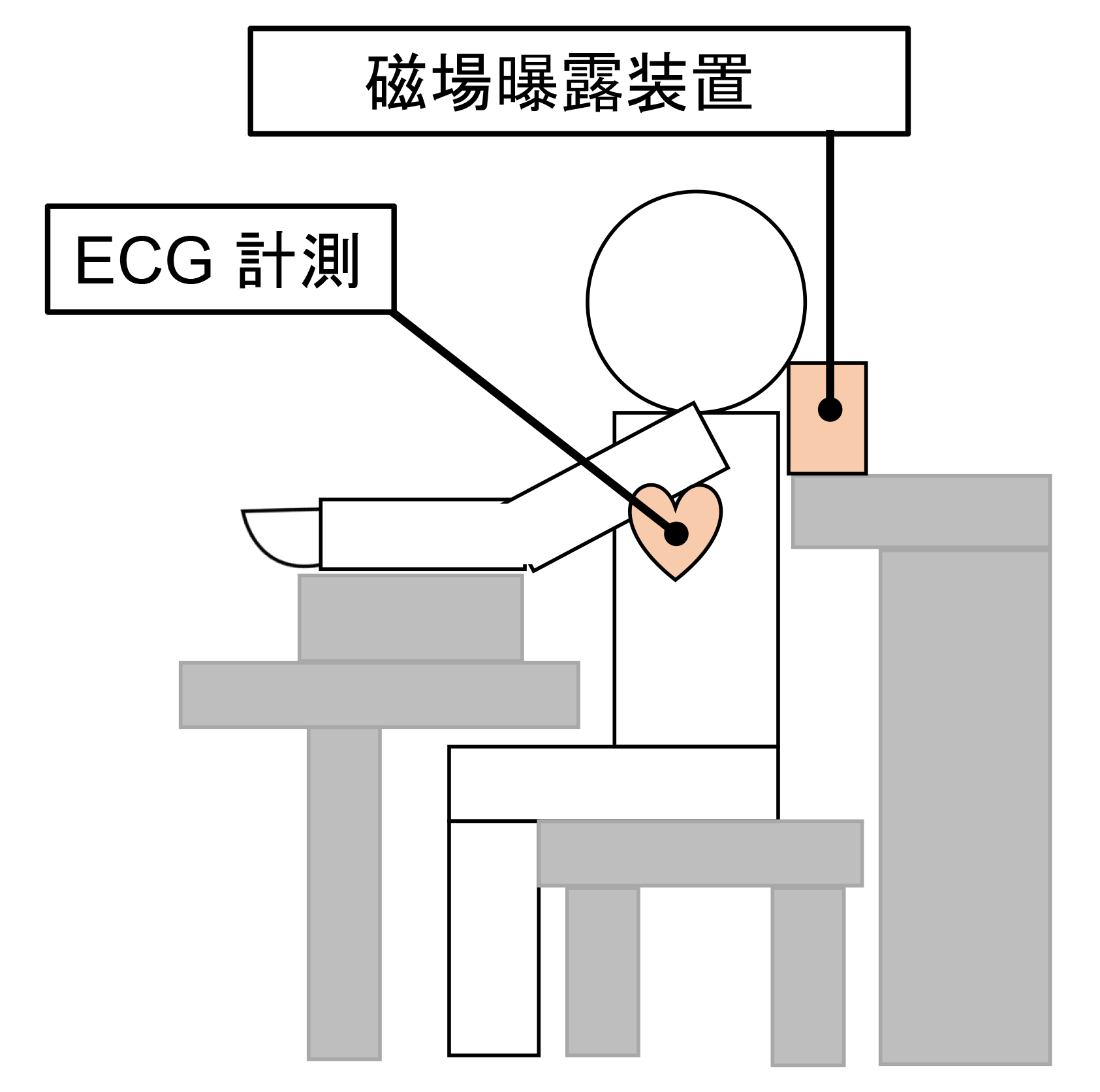


**ECG測定と頸部曝露**

**FMD**

血流依存性血管拡張反応（Flow-Mediated Dilatation: FMD）は，血管内皮機能とも呼ばれる血管の機能を非侵襲的に評価するためによく利用されている．FMD検査は，圧力が制御され高速に膨張するカフを用いて腕を駆血した後の血流増大によるずり応力により，血管内皮から血管拡張物資である一酸化窒素（Nitric Oxide: NO）が放出される．これに反応して起きる血管の拡張量を計測し，血管径変化の比率として算出することで血管内皮の機能を調べる．血管内皮機能が低下している場合，NOの産生が減少し，FMD値は低下する．これらを式で表すと以下のようになる．


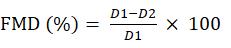
 (8)

ここで，*D*1 = 血管径のベースライン値，*D*2 = カフ解放 (減圧) 後に到達した血管径の最大値．このように，FMD 値は，血管径のベースライン値から最大値への変化率（％）として自動的に算出された．

本研究で使用したFMD計測装置は，下図のUNEXEF 18VG（UNEX株式会社, 名古屋, 日本）である．本装置の特徴として，長軸画像と2つの短軸画像を同時に抽出できる特殊なH型の超音波プローブが装備されている．そのため，血管走行と超音波ビームが直行となる位置に超音波プローブを調整することが容易である．さらに，血管位置がずれた際にもどちらの方向に動いたかが分かり，自動的に血管を見失うことがなく追跡できるといった特徴がある．


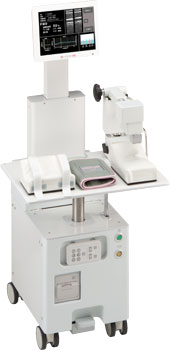


FMD計測装置


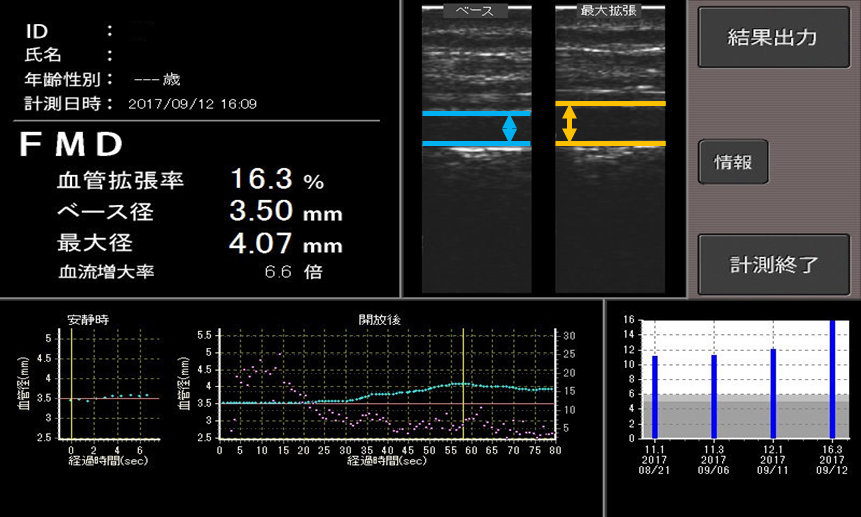


**FMDの測定結果**

計測前，被験者はベットの上で仰臥位の姿勢で左腕上腕部を磁気曝露装置の上に乗せ，前腕部にはFMD計測用のカフを装着した．5分間以上安静状態をとり，その状態で上腕背側に30分間磁場を曝露したが，FMD計測はその曝露前後で行った．磁場曝露を30分間に設定した理由は，FMD計測では計測部位にカフ圧をかけて測定するため，そのカフ圧負荷の影響を除くために測定間隔を30分以上あける必要があるためである．超音波プローブによる計測位置は被験者の腕の長さに応じて，左上腕動脈の肘から近位中枢側に50～80 mmの位置とし，磁場曝露もその血管の直下になるように調整した．計測位置調整後の計測は全自動であるが，1回の計測には約7～10分間を要する．すなわち，安静時のベースラインとなる動脈径を計測後に5分間の駆血を行い，駆血解放後の2分間連続的に動脈径を計測し，その最大径からFMD値が算出される．FMD計測において，前腕駆血と上腕駆血の2通りあるが，本実験ではNO依存性の高い前腕駆血で計測を行った．


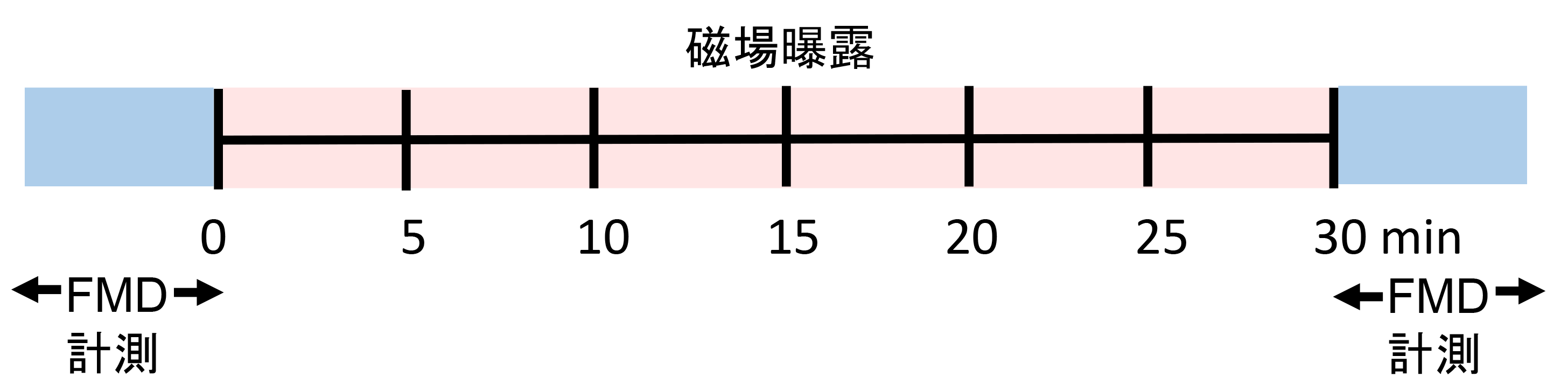


実験タイムプロトコル


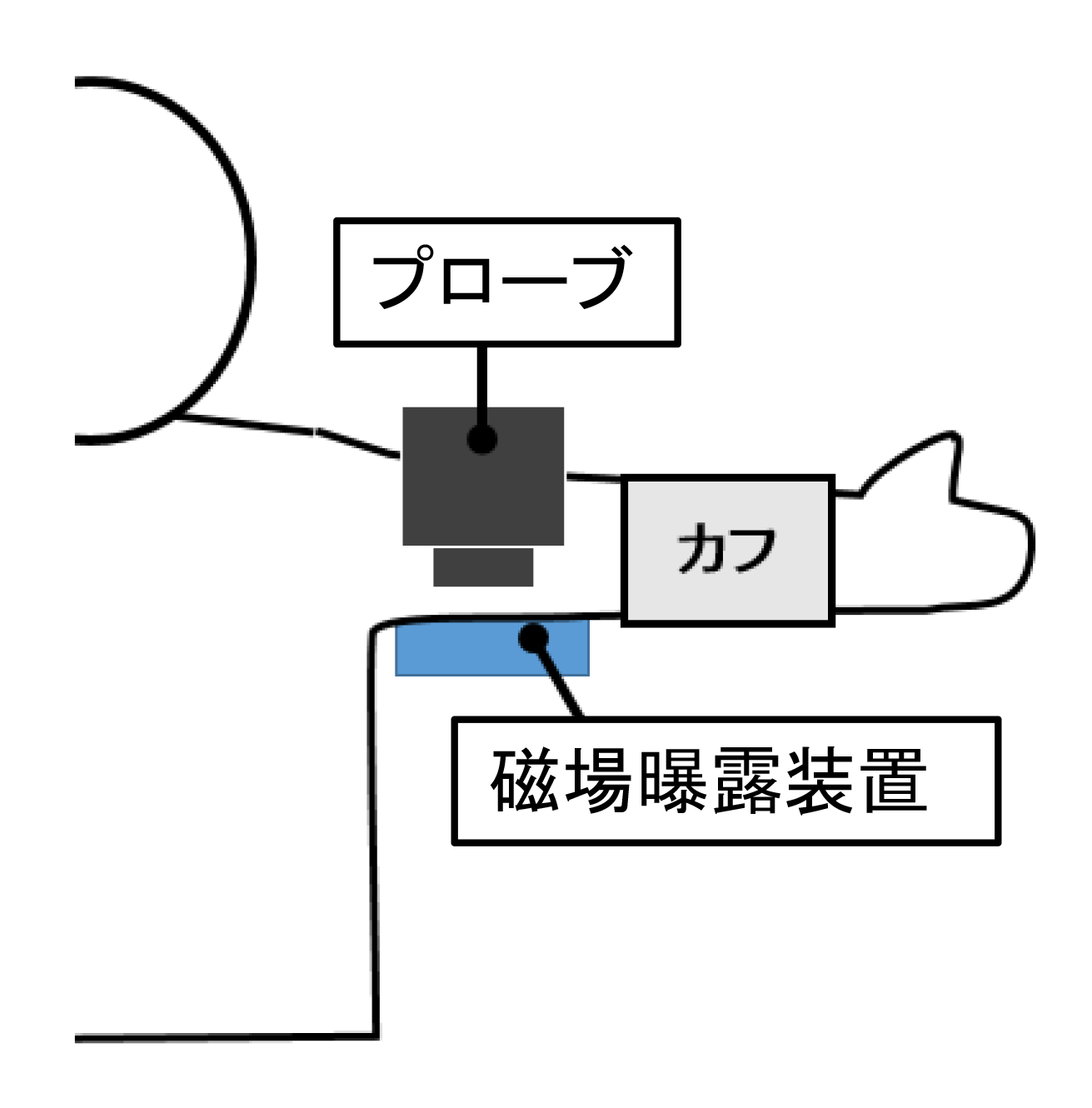


**FMD測定と上腕部曝露**
