# Supplementary material for: A 50 Hz magnetic field affects hemodynamics, ECG and vascular endothelial function in healthy adults: A pilot randomized controlled trial
Source: PLoS One. 2021 Aug 5;16(8):e0255242. doi: 10.1371/journal.pone.0255242 (PMC8341886; doi:10.1371/journal.pone.0255242)
Supplement: S4 File — (DOCX) [file pone.0255242.s004.docx]

**Table A.** **The Shapiro-Wilk test results for normality assumptions.**

**1. Subject baseline characteristics in the protocols A and B.**

| **Measured outcomes** | **Protocol A** | | **Protocol B** | |
| --- | --- | --- | --- | --- |
|  | **Statistic** | ***p*** | **Statistic** | ***p*** |
| Age (y.o.) | 0.508974 | 0.0001*** | 0.86630 | 0.0236* |
| Height (cm) | 0.963026 | 0.8080 | 0.91795 | 0.1580 |
| Weight (kg) | 0.925176 | 0.3868 | 0.90275 | 0.0903 |
| BMI (kg/m^2^) | 0.914810 | 0.3023 | 0.87193 | 0.0289* |
| Systolic BP (mmHg) | 0.902493 | 0.2226 | 0.972565 | 0.8476 |
| Diastolic BP (mmHg) | 0.944309 | 0.5856 | 0.926558 | 0.2157 |
| HR (bpm) | 0.926522 | 0.3990 | 0.96607 | 0.7424 |
| BT(℃) | 0.869483 | 0.0943 | 0.866205 | 0.0235* |

BMI, body mass index. BP, blood pressure; HR, heart rate; BT, body temperature. **p* < 0.05, ****p* < 0.001.

**2. Comparison of change rate (%) of ulnar arterial blood flow velocity (BFV), blood pressure (BP), heart rate (HR), hemoglobin oxygenation index (HOI), RRI, HF, LF/HF and FMD between 50 Hz MF and sham exposures.**

| **Measured outcomes** | **Exposure** | ***t* (min)** | **Statistic** | ***p*** |
| --- | --- | --- | --- | --- |
| BFV-sham exposure | Sham | 0 | – | – |
|  |  | 5 | 0.863321 | 0.0800 |
|  |  | 10 | 0.960986 | 0.7846 |
|  |  | 15 | 0.951486 | 0.6708 |
|  |  | 20 | 0.957498 | 0.7433 |
|  |  | 25 | 0.876507 | 0.1135 |
| BFV-forearm exposure | MF | 0 | – | – |
|  |  | 5 | 0.944422 | 0.5869 |
|  |  | 10 | 0.951899 | 0.6758 |
|  |  | 15 | 0.896227 | 0.1898 |
|  |  | 20 | 0.780953 | 0.0089** |
|  |  | 25 | 0.925051 | 0.3857 |
| BFV-upper arm exposure | MF | 0 | – | – |
|  |  | 5 | 0.966954 | 0.8512 |
|  |  | 10 | 0.965975 | 0.8408 |
|  |  | 15 | 0.934084 | 0.4729 |
|  |  | 20 | 0.913602 | 0.2935 |
|  |  | 25 | 0.923227 | 0.3696 |
| BFV-neck exposure | MF | 0 | – | – |
|  |  | 5 | 0.701574 | 0.0011** |
|  |  | 10 | 0.922658 | 0.3647 |
|  |  | 15 | 0.959622 | 0.7686 |
|  |  | 20 | 0.901015 | 0.2145 |
|  |  | 25 | 0.919565 | 0.3390 |
| Systolic BP | Sham | Pre | 0.886658 | 0.1482 |
|  |  | During | 0.918686 | 0.3319 |
|  |  | Post | 0.895931 | 01884 |
|  | MF | Pre | 0.843289 | 0.0468* |
|  |  | During | 0.951340 | 0.6691 |
|  |  | Post | 0.801461 | 0.0153* |
| Diastolic BP | Sham | Pre | 0.814576 | 0.0217* |
|  |  | During | 0.766622 | 0.0061** |
|  |  | Post | 0.838821 | 0.0415* |
|  | MF | Pre | 0.882761 | 0.1338 |
|  |  | During | 0.882465 | 0.1328 |
|  |  | Post | 0.817466 | 0.0234* |
| HR | Sham | Pre | 0.991875 | 0.9982 |
|  |  | During | 0.920311 | 0.3451 |
|  |  | Post | 0.917528 | 0.3229 |
|  | MF | Pre | 0.944022 | 0.5822 |
|  |  | During | 0.887992 | 0.1534 |
|  |  | Post | 0.970386 | 0.8859 |
| HOI-CH-1 | Sham | 5 | – | – |
|  |  | 10 | 0.951948 | 0.6764 |
|  |  | 15 | 0.888582 | 0.1558 |
|  |  | 20 | 0.886735 | 0.1845 |
|  |  | 25 | 0.891410 | 0.1677 |
|  | MF | 5 | – | – |
|  |  | 10 | 0.933119 | 0.4630 |
|  |  | 15 | 0.959177 | 0.7633 |
|  |  | 20 | 0.840496 | 0.0435* |
|  |  | 25 | 0.880553 | 0.1263 |
| HOI-CH-2 | Sham | 5 | – | – |
|  |  | 10 | 0.893724 | 0.1780 |
|  |  | 15 | 0.795853 | 0.0132* |
|  |  | 20 | 0.65801 | 0.0004*** |
|  |  | 25 | 0.817831 | 0.0235* |
|  | MF | 5 | – | – |
|  |  | 10 | 0.931190 | 0.4436 |
|  |  | 15 | 0.958403 | 0.7541 |
|  |  | 20 | 0.872530 | 0.1022 |
|  |  | 25 | 0.9551 | 0.7145 |
| ECG-RRI | Sham | 0.0 | – | – |
|  |  | 0.5 | 0.870128 | 0.0959 |
|  |  | 1.0 | 0.959954 | 0.7725 |
|  |  | 1.5 | 0.935044 | 0.4829 |
|  |  | 2.0 | 0.916922 | 0.3180 |
|  |  | 2.5 | 0.923149 | 0.3690 |
|  |  | 3.0 | 0.960073 | 0.7739 |
|  |  | 3.5 | 0.954309 | 0.7049 |
|  |  | 4.0 | 0.931661 | 0.4483 |
|  |  | 4.5 | 0.982689 | 0.9750 |
|  |  | 5.0 | 0.923257 | 0.3699 |
|  |  | 5.5 | 0.941367 | 0.5518 |
|  |  | 6.0 | 0.97066 | 0.8886 |
|  |  | 6.5 | 0.941323 | 0.5513 |
|  |  | 7.0 | 0.957561 | 0.7441 |
|  |  | 7.5 | 0.960413 | 0.7779 |
|  |  | 8.0 | 0.970615 | 0.8881 |
|  |  | 8.5 | 0.946311 | 0.6090 |
|  |  | 9.0 | 0.827949 | 0.0310* |
|  |  | 9.5 | 0.910552 | 0.2723 |
|  |  | 10.0 | 0.982856 | 0.9757 |
|  |  | 10.5 | 0.953439 | 0.6944 |
|  |  | 11.0 | 0.920878 | 0.3497 |
|  |  | 11.5 | 0.874908 | 0.1088 |
|  |  | 12.0 | 0.925333 | 0.3882 |
|  |  | 12.5 | 0.944852 | 0.5919 |
|  |  | 13.0 | 0.970614 | 0.8881 |
|  |  | 13.5 | 0.972323 | 0.9040 |
|  |  | 14.0 | 0.921141 | 0.3519 |
|  |  | 14.5 | 0.939774 | 0.5340 |
|  |  | 15.0 | 0.890337 | 0.1631 |
|  |  | 15.5 | 0.850281 | 0.0565 |
|  |  | 16.0 | 0.953718 | 0.6978 |
|  |  | 16.5 | 0.865204 | 0.0841 |
|  |  | 17.0 | 0.975408 | 0.9299 |
|  |  | 17.5 | 0.927606 | 0.4091 |
|  |  | 18.0 | 0.941871 | 0.5575 |
|  |  | 18.5 | 0.852935 | 0.0606 |
|  |  | 19.0 | 0.795560 | 0.0131* |
|  |  | 19.5 | 0.899982 | 0.2089 |
|  |  | 20.0 | 0.843904 | 0.0476* |
|  |  | 20.5 | 0.926833 | 0.4019 |
|  |  | 21.0 | 0.945177 | 0.5957 |
|  |  | 21.5 | 0.805893 | 0.0172* |
|  |  | 22.0 | 0.929818 | 0.4301 |
|  |  | 22.5 | 0.936334 | 0.4965 |
|  |  | 23.0 | 0.919374 | 0.3375 |
|  |  | 23.5 | 0.871577 | 0.0997 |
|  |  | 24.0 | 0.849659 | 0.0555 |
|  |  | 24.5 | 0.874100 | 0.1065 |
|  |  | 25.0 | 0.874496 | 0.1077 |
|  | MF | 0.0 | – | – |
|  |  | 0.5 | 0.913050 | 0.2896 |
|  |  | 1.0 | 0.874888 | 0.1088 |
|  |  | 1.5 | 0.814436 | 0.0216* |
|  |  | 2.0 | 0.952254 | 0.6801 |
|  |  | 2.5 | 0.873953 | 0.1061 |
|  |  | 3.0 | 0.525738 | 0.0001*** |
|  |  | 3.5 | 0.869916 | 0.0954 |
|  |  | 4.0 | 0.869379 | 0.0940 |
|  |  | 4.5 | 0.905585 | 0.2407 |
|  |  | 5.0 | 0.936269 | 0.4958 |
|  |  | 5.5 | 0.984846 | 0.9836 |
|  |  | 6.0 | 0.957737 | 0.7462 |
|  |  | 6.5 | 0.921840 | 0.3578 |
|  |  | 7.0 | 0.936399 | 0.4972 |
|  |  | 7.5 | 0.940323 | 0.5401 |
|  |  | 8.0 | 0.967138 | 0.8532 |
|  |  | 8.5 | 0.917678 | 0.3240 |
|  |  | 9.0 | 0.938470 | 0.5196 |
|  |  | 9.5 | 0.897996 | 0.1986 |
|  |  | 10.0 | 0.956662 | 0.7333 |
|  |  | 10.5 | 0.931750 | 0.4492 |
|  |  | 11.0 | 0.967178 | 0.8536 |
|  |  | 11.5 | 0.913090 | 0.2899 |
|  |  | 12.0 | 0.963740 | 0.8161 |
|  |  | 12.5 | 0.983837 | 0.9799 |
|  |  | 13.0 | 0.909202 | 0.2634 |
|  |  | 13.5 | 0.949449 | 0.6463 |
|  |  | 14.0 | 0.909352 | 0.2644 |
|  |  | 14.5 | 0.949576 | 0.6479 |
|  |  | 15.0 | 0.967713 | 0.8592 |
|  |  | 15.5 | 0.937545 | 0.5095 |
|  |  | 16.0 | 0.809278 | 0.0188* |
|  |  | 16.5 | 0.928650 | 0.4189 |
|  |  | 17.0 | 0.916548 | 0.3153 |
|  |  | 17.5 | 0.886024 | 0.1458 |
|  |  | 18.0 | 0.867870 | 0.0903 |
|  |  | 18.5 | 0.916623 | 0.3159 |
|  |  | 19.0 | 0.932038 | 0.4520 |
|  |  | 19.5 | 0.925933 | 0.3937 |
|  |  | 20.0 | 0.905324 | 0.2391 |
|  |  | 20.5 | 0.943443 | 0.5755 |
|  |  | 21.0 | 0.932736 | 0.4591 |
|  |  | 21.5 | 0.901691 | 0.2182 |
|  |  | 22.0 | 0.883247 | 0.1355 |
|  |  | 22.5 | 0.946226 | 0.6080 |
|  |  | 23.0 | 0.937727 | 0.5115 |
|  |  | 23.5 | 0.955943 | 0.7246 |
|  |  | 24.0 | 0.939470 | 0.5306 |
|  |  | 24.5 | 0.932330 | 0.4550 |
|  |  | 25.0 | 0.943398 | 0.5750 |
| ECG-HF | Sham | 0.0 | – | – |
|  |  | 0.5 | 0.940363 | 0.5406 |
|  |  | 1.0 | 0.933327 | 0.4651 |
|  |  | 1.5 | 0.952586 | 0.6841 |
|  |  | 2.0 | 0.923929 | 0.3758 |
|  |  | 2.5 | 0.880284 | 0.1254 |
|  |  | 3.0 | 0.946694 | 0.6135 |
|  |  | 3.5 | 0.907079 | 0.2498 |
|  |  | 4.0 | 0.923611 | 0.3730 |
|  |  | 4.5 | 0.928162 | 0.4143 |
|  |  | 5.0 | 0.896810 | 0.1927 |
|  |  | 5.5 | 0.924427 | 0.3802 |
|  |  | 6.0 | 0.972987 | 0.9098 |
|  |  | 6.5 | 0.934785 | 0.4802 |
|  |  | 7.0 | 0.946612 | 0.6125 |
|  |  | 7.5 | 0.973686 | 0.9159 |
|  |  | 8.0 | 0.956705 | 0.7338 |
|  |  | 8.5 | 0.961335 | 0.7886 |
|  |  | 9.0 | 0.958507 | 0.7554 |
|  |  | 9.5 | 0.955133 | 0.7149 |
|  |  | 10.0 | 0.873922 | 0.1060 |
|  |  | 10.5 | 0.956117 | 0.7267 |
|  |  | 11.0 | 0.952273 | 0.6803 |
|  |  | 11.5 | 0.925920 | 0.3935 |
|  |  | 12.0 | 0.971341 | 0.8950 |
|  |  | 12.5 | 0.975403 | 0.9298 |
|  |  | 13.0 | 0.913314 | 0.2915 |
|  |  | 13.5 | 0.920285 | 0.3449 |
|  |  | 14.0 | 0.928059 | 0.4133 |
|  |  | 14.5 | 0.946895 | 0.6159 |
|  |  | 15.0 | 0.892397 | 0.1720 |
|  |  | 15.5 | 0.963057 | 0.8084 |
|  |  | 16.0 | 0.967020 | 0.8519 |
|  |  | 16.5 | 0.936579 | 0.4991 |
|  |  | 17.0 | 0.966222 | 0.8434 |
|  |  | 17.5 | 0.912486 | 0.2856 |
|  |  | 18.0 | 0.936389 | 0.4971 |
|  |  | 18.5 | 0.939748 | 0.5337 |
|  |  | 19.0 | 0.936778 | 0.5013 |
|  |  | 19.5 | 0.921865 | 0.3580 |
|  |  | 20.0 | 0.913627 | 0.2937 |
|  |  | 20.5 | 0.916474 | 0.3148 |
|  |  | 21.0 | 0.923989 | 0.3763 |
|  |  | 21.5 | 0.950761 | 0.6621 |
|  |  | 22.0 | 0.909233 | 0.2636 |
|  |  | 22.5 | 0.865576 | 0.0850 |
|  |  | 23.0 | 0.944263 | 0.5850 |
|  |  | 23.5 | 0.883985 | 0.1382 |
|  |  | 24.0 | 0.940298 | 0.5398 |
|  |  | 24.5 | 0.927612 | 0.4091 |
|  |  | 25.0 | 0.879681 | 0.1234 |
|  | MF | 0.0 | – | – |
|  |  | 0.5 | 0.934388 | 0.4760 |
|  |  | 1.0 | 0.951996 | 0.6770 |
|  |  | 1.5 | 0.838917 | 0.0417* |
|  |  | 2.0 | 0.880117 | 0.1249 |
|  |  | 2.5 | 0.915253 | 0.3056 |
|  |  | 3.0 | 0.682783 | 0.0007*** |
|  |  | 3.5 | 0.924925 | 0.3846 |
|  |  | 4.0 | 0.828566 | 0.0316* |
|  |  | 4.5 | 0.854677 | 0.0635 |
|  |  | 5.0 | 0.891539 | 0.1682 |
|  |  | 5.5 | 0.964058 | 0.8197 |
|  |  | 6.0 | 0.855866 | 0.0656 |
|  |  | 6.5 | 0.695285 | 0.0010** |
|  |  | 7.0 | 0.845976 | 0.0503 |
|  |  | 7.5 | 0.901252 | 0.2158 |
|  |  | 8.0 | 0.928960 | 0.4219 |
|  |  | 8.5 | 0.939396 | 0.5298 |
|  |  | 9.0 | 0.964806 | 0.8280 |
|  |  | 9.5 | 0.820728 | 0.0256* |
|  |  | 10.0 | 0.938804 | 0.5233 |
|  |  | 10.5 | 0.810480 | 0.0194* |
|  |  | 11.0 | 0.952199 | 0.6794 |
|  |  | 11.5 | 0.933455 | 0.4664 |
|  |  | 12.0 | 0.739942 | 0.0030** |
|  |  | 12.5 | 0.944653 | 0.5896 |
|  |  | 13.0 | 0.811167 | 0.0198* |
|  |  | 13.5 | 0.790300 | 0.0114* |
|  |  | 14.0 | 0.847837 | 0.0529 |
|  |  | 14.5 | 0.934750 | 0.4798 |
|  |  | 15.0 | 0.782908 | 0.0093** |
|  |  | 15.5 | 0.861567 | 0.0764 |
|  |  | 16.0 | 0.934475 | 0.4769 |
|  |  | 16.5 | 0.900699 | 0.2127 |
|  |  | 17.0 | 0.739477 | 0.0030** |
|  |  | 17.5 | 0.916084 | 0.3118 |
|  |  | 18.0 | 0.863700 | 0.0808 |
|  |  | 18.5 | 0.973175 | 0.9115 |
|  |  | 19.0 | 0.905796 | 0.2419 |
|  |  | 19.5 | 0.874458 | 0.1076 |
|  |  | 20.0 | 0.927788 | 0.4108 |
|  |  | 20.5 | 0.948463 | 0.6345 |
|  |  | 21.0 | 0.891729 | 0.1690 |
|  |  | 21.5 | 0.884028 | 0.1383 |
|  |  | 22.0 | 0.891857 | 0.1696 |
|  |  | 22.5 | 0.870695 | 0.0974 |
|  |  | 23.0 | 0.933364 | 0.4655 |
|  |  | 23.5 | 0.729839 | 0.0023** |
|  |  | 24.0 | 0.935706 | 0.4899 |
|  |  | 24.5 | 0.950556 | 0.6596 |
|  |  | 25.0 | 0.838200 | 0.0409* |
| ECG-LF/HF | Sham | 0.0 | – | – |
|  |  | 0.5 | 0.872978 | 0.1034 |
|  |  | 1.0 | 0.946398 | 0.6100 |
|  |  | 1.5 | 0.836237 | 0.0388* |
|  |  | 2.0 | 0.965206 | 0.8324 |
|  |  | 2.5 | 0.918050 | 0.3269 |
|  |  | 3.0 | 0.895609 | 0.1868 |
|  |  | 3.5 | 0.938587 | 0.5209 |
|  |  | 4.0 | 0.892324 | 0.1717 |
|  |  | 4.5 | 0.904070 | 0.2317 |
|  |  | 5.0 | 0.866935 | 0.0881 |
|  |  | 5.5 | 0.912498 | 0.2857 |
|  |  | 6.0 | 0.921438 | 0.3544 |
|  |  | 6.5 | 0.914253 | 0.2982 |
|  |  | 7.0 | 0.925329 | 0.3882 |
|  |  | 7.5 | 0.884707 | 0.1408 |
|  |  | 8.0 | 0.593644 | 0.0001*** |
|  |  | 8.5 | 0.860236 | 0.0737 |
|  |  | 9.0 | 0.912445 | 0.2853 |
|  |  | 9.5 | 0.977421 | 0.9447 |
|  |  | 10.0 | 0.874458 | 0.1076 |
|  |  | 10.5 | 0.819320 | 0.0246* |
|  |  | 11.0 | 0.863428 | 0.0803 |
|  |  | 11.5 | 0.903830 | 0.2303 |
|  |  | 12.0 | 0.846591 | 0.0512 |
|  |  | 12.5 | 0.906032 | 0.2434 |
|  |  | 13.0 | 0.940062 | 0.5372 |
|  |  | 13.5 | 0.888330 | 0.1548 |
|  |  | 14.0 | 0.888878 | 0.1570 |
|  |  | 14.5 | 0.908508 | 0.2589 |
|  |  | 15.0 | 0.838640 | 0.0413* |
|  |  | 15.5 | 0.955065 | 0.7141 |
|  |  | 16.0 | 0.818402 | 0.0240* |
|  |  | 16.5 | 0.915241 | 0.3055 |
|  |  | 17.0 | 0.789604 | 0.0112* |
|  |  | 17.5 | 0.884408 | 0.1397 |
|  |  | 18.0 | 0.737787 | 0.0029** |
|  |  | 18.5 | 0.756636 | 0.0047** |
|  |  | 19.0 | 0.944005 | 0.5820 |
|  |  | 19.5 | 0.903300 | 0.2272 |
|  |  | 20.0 | 0.883934 | 0.1380 |
|  |  | 20.5 | 0.934652 | 0.4788 |
|  |  | 21.0 | 0.937931 | 0.5137 |
|  |  | 21.5 | 0.910572 | 0.2725 |
|  |  | 22.0 | 0.925009 | 0.3853 |
|  |  | 22.5 | 0.899547 | 0.2066 |
|  |  | 23.0 | 0.938009 | 0.5146 |
|  |  | 23.5 | 0.892079 | 0.1706 |
|  |  | 24.0 | 0.938657 | 0.5216 |
|  |  | 24.5 | 0.909945 | 0.2683 |
|  |  | 25.0 | 0.817557 | 0.0235* |
|  | MF | 0.0 | – | – |
|  |  | 0.5 | 0.872440 | 0.1020 |
|  |  | 1.0 | 0.792636 | 0.0121* |
|  |  | 1.5 | 0.809347 | 0.0189* |
|  |  | 2.0 | 0.782247 | 0.0092** |
|  |  | 2.5 | 0.828551 | 0.0315* |
|  |  | 3.0 | 0.785447 | 0.0100* |
|  |  | 3.5 | 0.941634 | 0.5549 |
|  |  | 4.0 | 0.904706 | 0.2354 |
|  |  | 4.5 | 0.906668 | 0.2473 |
|  |  | 5.0 | 0.862133 | 0.0775 |
|  |  | 5.5 | 0.878285 | 0.1190 |
|  |  | 6.0 | 0.779436 | 0.0085** |
|  |  | 6.5 | 0.826554 | 0.0299* |
|  |  | 7.0 | 0.777860 | 0.0082** |
|  |  | 7.5 | 0.926745 | 0.4011 |
|  |  | 8.0 | 0.817171 | 0.0233* |
|  |  | 8.5 | 0.856079 | 0.0660 |
|  |  | 9.0 | 0.896602 | 0.1916 |
|  |  | 9.5 | 0.875342 | 0.1101 |
|  |  | 10.0 | 0.943760 | 0.5792 |
|  |  | 10.5 | 0.859866 | 0.0730 |
|  |  | 11.0 | 0.943050 | 0.5710 |
|  |  | 11.5 | 0.747274 | 0.0037** |
|  |  | 12.0 | 0.855708 | 0.0653 |
|  |  | 12.5 | 0.800159 | 0.0148* |
|  |  | 13.0 | 0.778404 | 0.0083** |
|  |  | 13.5 | 0.880598 | 0.1265 |
|  |  | 14.0 | 0.937631 | 0.5105 |
|  |  | 14.5 | 0.784713 | 0.0098** |
|  |  | 15.0 | 0.662689 | 0.0004*** |
|  |  | 15.5 | 0.843090 | 0.0466* |
|  |  | 16.0 | 0.872743 | 0.1028 |
|  |  | 16.5 | 0.986213 | 0.9880 |
|  |  | 17.0 | 0.704040 | 0.0012** |
|  |  | 17.5 | 0.878933 | 0.1210 |
|  |  | 18.0 | 0.766648 | 0.0061** |
|  |  | 18.5 | 0.916854 | 0.3177 |
|  |  | 19.0 | 0.812609 | 0.0206* |
|  |  | 19.5 | 0.912480 | 0.2856 |
|  |  | 20.0 | 0.855362 | 0.0647 |
|  |  | 20.5 | 0.933977 | 0.4718 |
|  |  | 21.0 | 0.901230 | 0.2156 |
|  |  | 21.5 | 0.987927 | 0.9924 |
|  |  | 22.0 | 0.954100 | 0.7024 |
|  |  | 22.5 | 0.750419 | 0.0040** |
|  |  | 23.0 | 0.865812 | 0.0855 |
|  |  | 23.5 | 0.886010 | 0.1457 |
|  |  | 24.0 | 0.897758 | 0.1970 |
|  |  | 24.5 | 0.944081 | 0.5829 |
|  |  | 25.0 | 0.894161 | 0.1800 |
| FMD | Sham | 0 | 0.979456 | 0.9362 |
|  |  | 30 | 0.979681 | 0.9385 |
|  | MF | 0 | 0.919152 | 0.1651 |
|  |  | 30 | 0.969018 | 0.7917 |

BFV, blood flow velocity; BP, blood pressure; HR, heart rate; HOI, hemoglobin oxygenation index; RRI, R-R interval; HF, high-frequency; LF, low-frequency; FMD, flow-mediated dilation. **p* < 0.05, ***p* < 0.01, ****p* < 0.001.


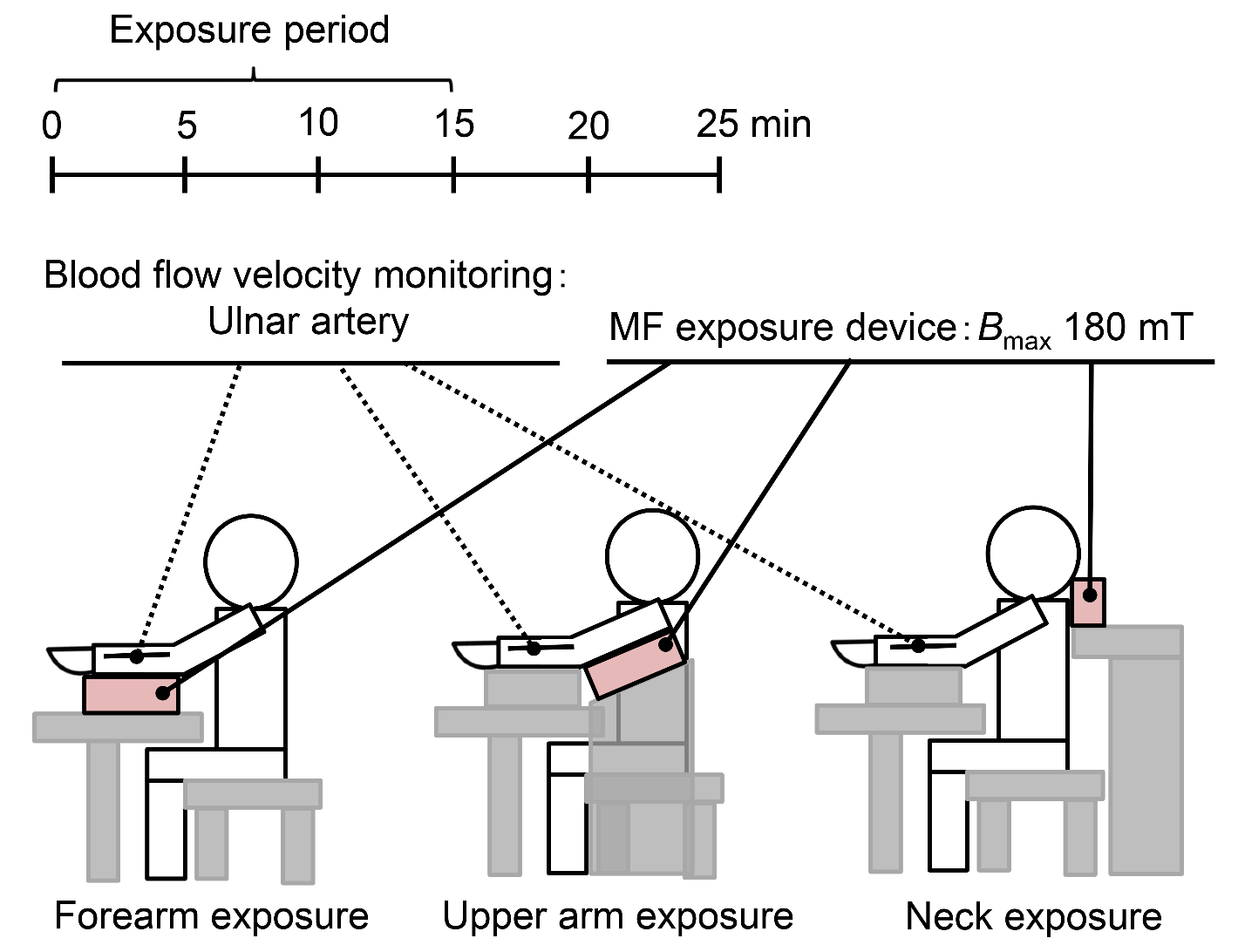


**Fig A. The measurement of blood flow velocity under three different regional exposures to a 50 Hz MF.** The forearm exposure, upper arm exposure or neck exposure to a 50 Hz MF and the measurement of ulnar artery blood flow velocity. All experiments were performed with the subject in the same sitting posture. The blood flow velocity was measured at 5-min intervals for 25 min. The MF or sham control exposure was performed continuously for 15 min.


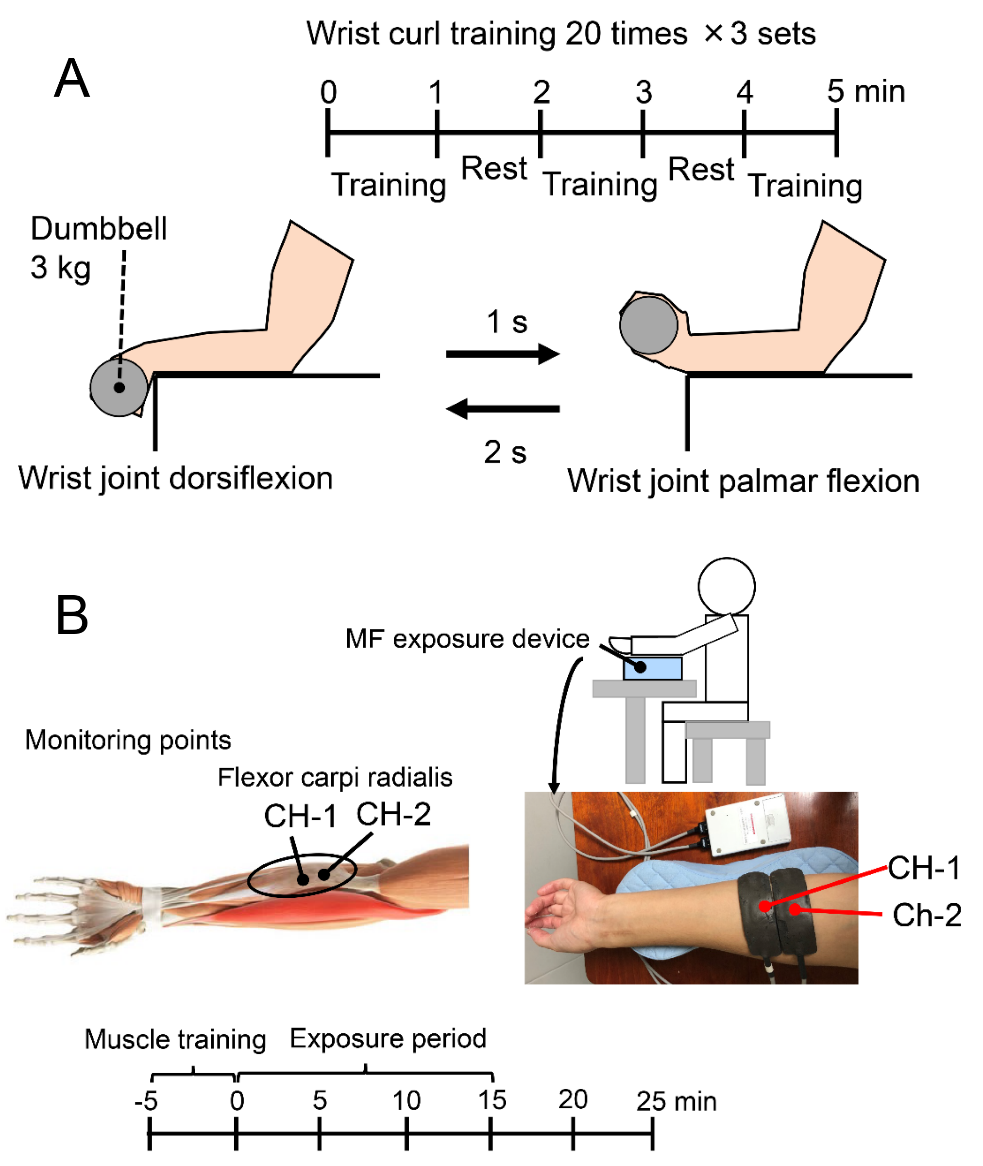


**Fig B. The fNIRS measurement of the ventral side of the forearm muscle under the forearm exposure to a 50 Hz MF after cessation of the muscle loading exercise.** (A) protocol of muscle loading exercise. (B) MF exposure and monitoring of the muscle hemodynamic changes of the left forearm after muscle loading exercise. CH-1: Middle part of flexor carpi radialis muscles. CH-2: Proximal part of flexor carpi radialis muscles.


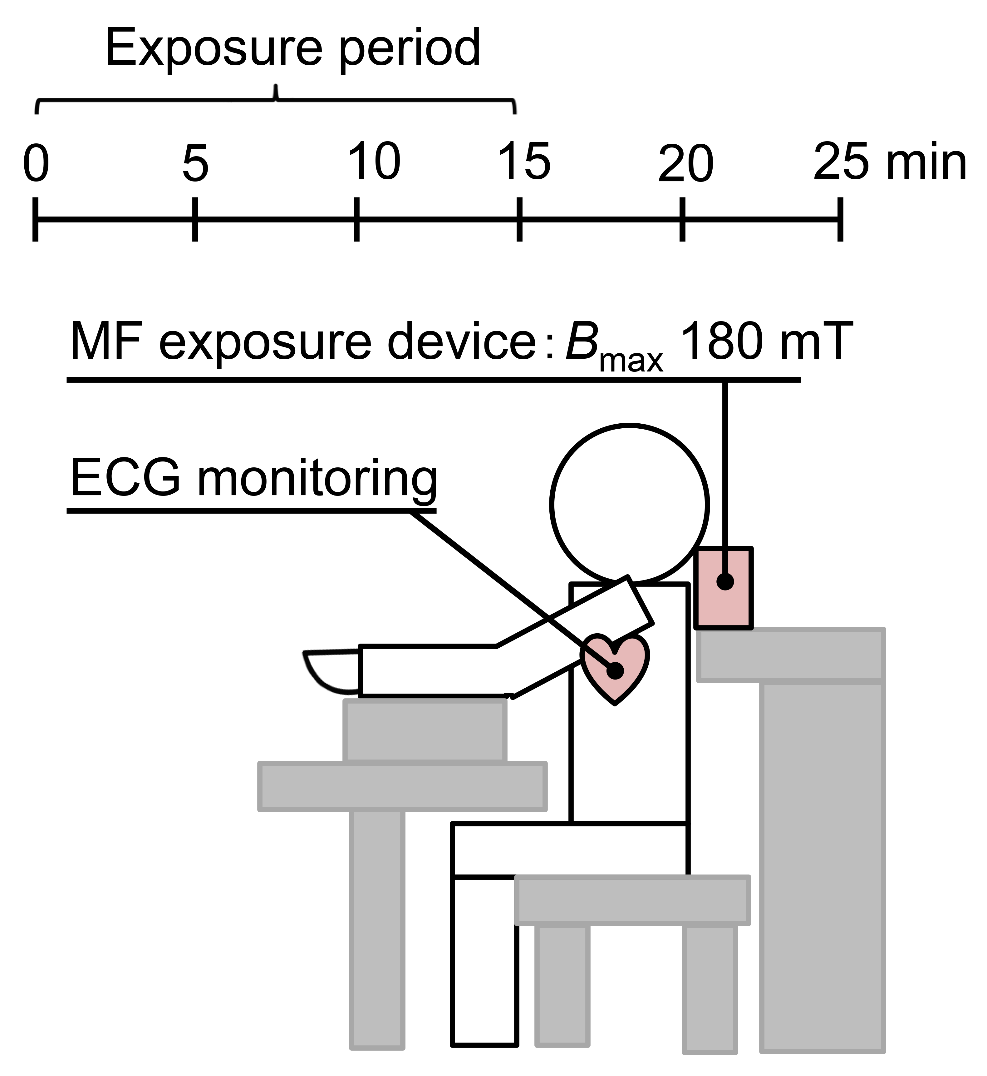


**Fig C. The ECG monitoring under the neck exposure to a 50 Hz MF.** All experiments were performed with the subject in the sitting posture. The ECG was monitored continuously for 25 min. The MF or sham control exposure was performed continuously for 15 min.


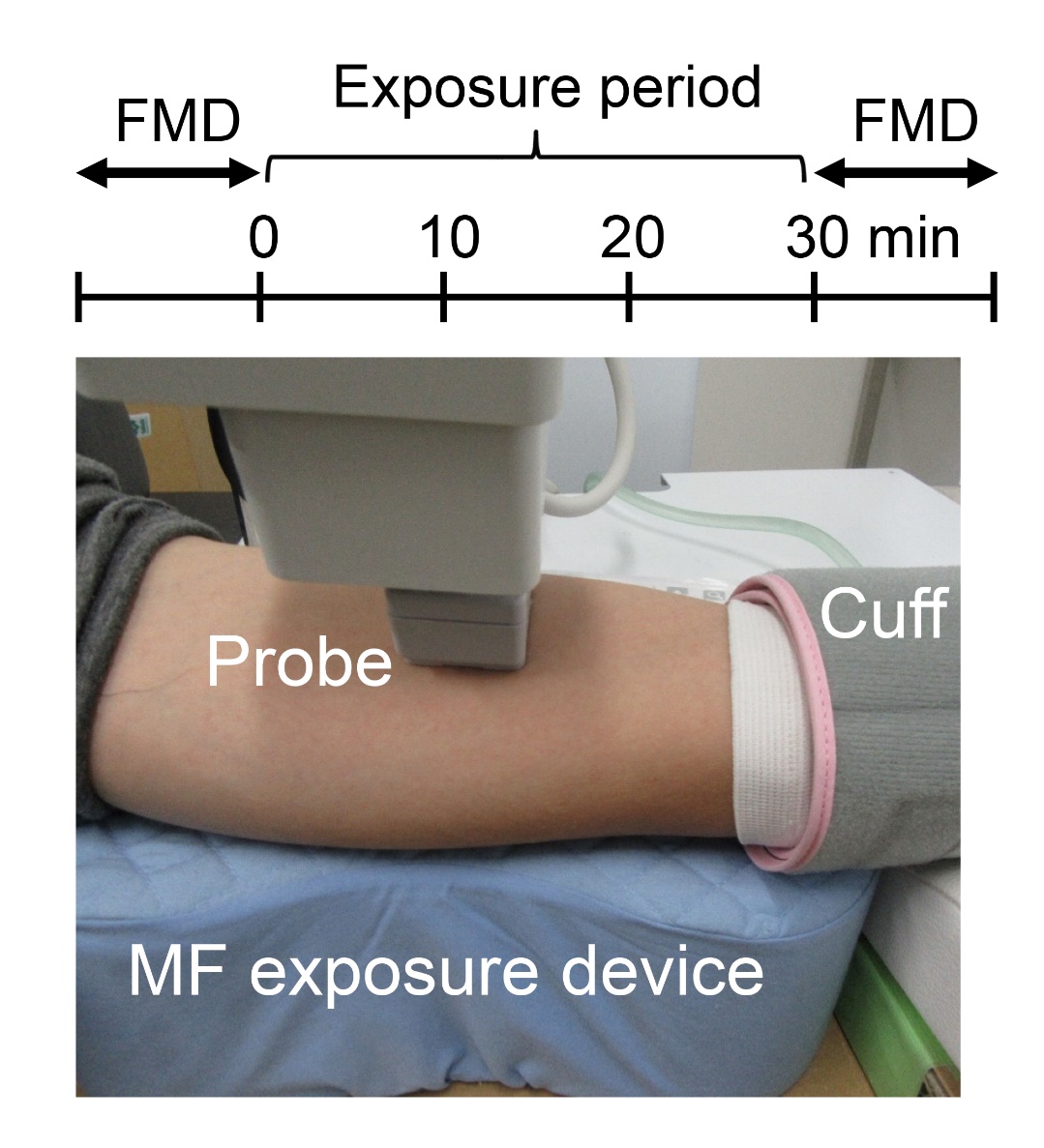


**Fig D. The FMD measurement.** All experiments were performed with the subject lying on a bed in the supine position. The left brachial artery FMD was measured during the pre-exposure and post-exposure periods. The MF or sham control exposure in the upper arm was performed continuously for 30 min.


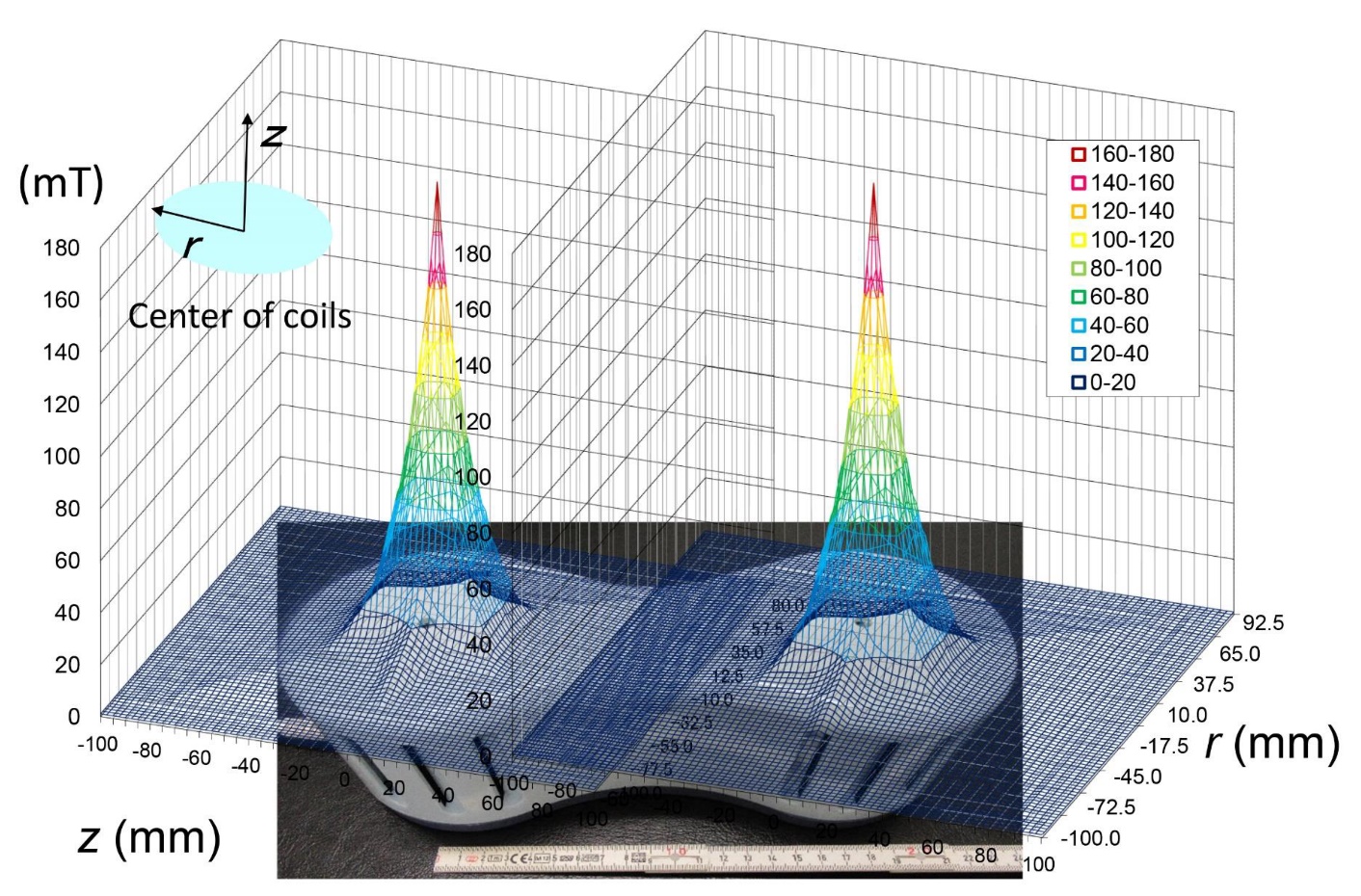


**Fig E. Spatial distribution of *B*_max_ values in a 50 Hz MF exposure device.** Two separate electromagnetic coils are set horizontally inside the MF exposure device and the value of *B*_max_ is 180 mT on the surface of the MF exposure device above the center of the double induction coils.


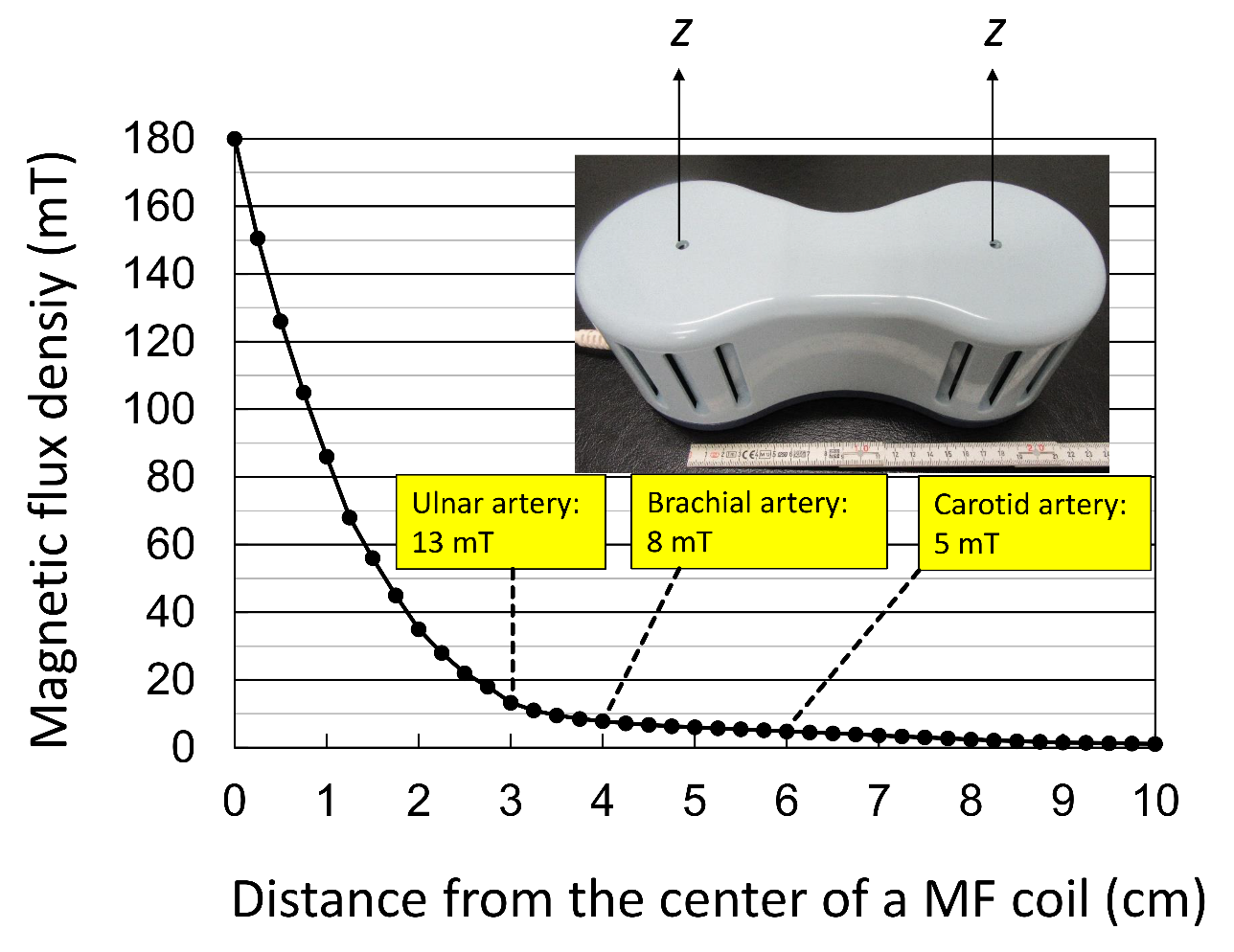


**Fig F. The estimated *B*_max_ values in three different regional exposures to a 50 Hz MF.** The *B*_max_ values in an ulnar artery, a brachial artery, and a carotid artery are approximately 13 mT, 8 mT, and 5 mT, in which the distances from the surface *B*_max_ 180 mT of the MF exposure device are approximately 3 cm, 4 cm, and 6 cm, respectively.
