# Supplementary material for: A 50 Hz magnetic field affects hemodynamics, ECG and vascular endothelial function in healthy adults: A pilot randomized controlled trial
Source: PLoS One. 2021 Aug 5;16(8):e0255242. doi: 10.1371/journal.pone.0255242 (PMC8341886; doi:10.1371/journal.pone.0255242)
Supplement: Attachment — Submitted filename: PO-journal-Response to Reviewer-21-03-02.docx [file pone.0255242.s006.docx]

Reviewers’ comments are italicized:

Reviewer #3:

*I come to this revised manuscript as a new reviewer.*

*This appears to be a crossover trial of 50Hz EM field. There are issues here in that the randomization appears to have taken place between patients to decide what was measured; in other words there are two crossover trials.*

*Patients were initially randomized between the two trials - this is not that important as these are two trials; but the last 6 were allocated to trial 2. The fact that the sample size changed is important. The sample size calculation is entirely opaque here, and there is no reasonable discussion of why it was not sorted up front - it at present appears it might have been affected by interim data, which would imply that there is a penalty to pay statistically, and this is not mentioned.*

*Where randomization is important is in allocating the order of therapies - here we have a 4 period design with either ABAB or BABA - this is not the best 4 period design, but needs to be analyzed as a crossover trial. At present this is not being done as we see differences between groups (i.e. unpaired analyses) and not between periods within a patient (paired data). It is unreasonable to perform an sd on 2 observations as given. The issue of time by treatment interactions and period effects are missing.*

*At present therefore, this trial report fails to justify the sample size and in particular the adjustment, clarify the randomization within trial, nor to use the correct analysis and graphical methods that account for crossover. Beyond this the paper is impossible to review as these factors may entirely change the message.*

Authors’ response in blue:

**The sample size calculation:**

Please see the methods, results, and discussion together with Table 2, Figs 3 and 6:

To detect a statistical difference in the mean change from baseline between real magnetic field (MF) and sham control exposures, we estimated the magnitude of the MF effects on blood flow velocity by setting SD = 10 cm/s and power = 0.80 using a statistical software package (EZR version 1.41, Saitama Medical Center, Jichi Medical University, Saitama, Japan), which is available online (http://www.jichi.ac.jp/saitama-sct/SaitamaHP.files/statmedEN.html). The results of sample size requirements are shown in Table 2.

**Table 2. Sample size required to determine significant 50 Hz MF effects on blood flow velocity (BFV).**

| **BFV difference (cm/s)** | **No. of subjects** |
| --- | --- |
| 5.0 | 63 |
| 6.0 | 44 |
| 7.0 | 33 |
| 8.0 | 25 |
| 9.0 | 20 |
| 10.0 | 16 |
| 11.0 | 13 |
| 12.0 | 11 |
| 13.0 | 10 |
| 14.0 | 9 |
| 15.0 | 7 |

Our obtained results of mean (SD) values of blood flow velocity differences between both conditions for 15-min exposure are 22.2% (9.4%), 12.7% (6.0%), and 11.6% (8.5%) in Fig 3A, B, and C, respectively. In these cases, the re-estimated sample size required to determine significant MF effects on blood flow velocity is at least 9 subjects. For the primary outcome measure, the re-estimated minimum sample size of 10 subjects would be able to detect significant differences between both conditions. In the case of FMD of 16 subjects for protocol B, mean (SD) values of FMD differences between both conditions were 1.59% (1.45%) for 30-min exposure in Fig 5. Here, the estimated minimum sample size is calculated to be 14 subjects per each condition by setting power = 0.80 using the EZR software package. Therefore, we estimated that the total sample size of 16 subjects would be large enough to detect significant MF effects on FMD. We cannot rule out the possibility that differences between the MF and sham exposures may have become more significant if more subjects were studied.

**Allocating the order of therapies as a crossover trial:**

Please see the methods:

Randomization was performed according to our preliminary trial protocol [50,51]. Briefly, all participants were divided evenly into two conditions with an allocation ratio 1:1 after block randomization with a permuted block size of 2 according to computer-generated random numbers by H.O. Thus, the allocation of which condition would receive real MFs for the first time was randomly generated. Thereafter, allocation of crossover sequence was done; ABAB or BABA.

**The issue of time by treatment interactions and period effects are missing:**

Please see the methods and results:

Statistical analyses were performed using SAS Analytics version 9.4 (SAS Institute, Cary, NC, USA). A two-way repeated-measures ANOVA was calculated with the factors “Time” and “Condition”.

Fig 3: Effects of MF exposure on blood flow velocity

In the forearm exposure, the repeated-measures ANOVA showed a significant Condition by Time interaction [*F*(5, 90) = 21.51, *p* < 0.0001]. The MF exposure significantly increased blood flow velocity values during the MF exposure period, and the blood flow velocity value peaked at 10 min after the start of the exposure, increasing from the baseline value by 13.4% (Fig 3A). After the termination of the exposure, the blood flow velocity gradually returned to the baseline value (Fig 3A).

In addition, in the upper arm exposure, the repeated-measures ANOVA showed a significant Condition by Time interaction [*F*(5, 90) = 11.60, *p* < 0.0001]. The MF exposure significantly increased blood flow velocity values during the MF exposure period, and the value of blood flow velocity peaked at 5-min, increasing from the baseline value by 4.6% (Fig 3B).

In the neck exposure, the repeated-measures ANOVA showed a significant Condition by Time interaction [*F*(5, 90) = 11.82, *p* < 0.0001]. No significant change in blood flow velocity values was induced by the MF exposure, but there were significant differences between the MF and sham exposures (Fig 3C).

Table 3: Effects of MF exposure on blood pressure

The repeated-measures ANOVA of BP showed no significant Condition by Time interaction [*F*(2, 36) = 1.34, *p* = 0.2750] and [*F*(2, 36) = 1.78, *p* = 0.1826] in systolic and diastolic BP, respectively. In the MF and sham exposures, both blood pressure values were not significantly changed from the pre (baseline) values, and there was no significant difference between both conditions (Table 3). In addition, the repeated-measures ANOVA of heart rate showed no significant Condition by Time interaction [*F*(2, 36) = 0.56, *p* = 0.5746]. In the MF and sham exposures, the heart rate values were not significantly changed from the pre (baseline) values and there was no significant difference between both conditions (Table 3).

Fig 4: Effects of MF exposure on hemoglobin oxygenation

The repeated-measures ANOVA of hemoglobin oxygenation index showed no significant Condition by Time interaction [*F*(4, 72) = 1.60, *p* = 0.1834] in ch 1, and [*F*(4, 72) = 1.02, *p* = 0.4042] in ch 2. MF exposure showed faster and higher decrease (recovery) rates of hemoglobin oxygenation index values than those of sham exposure from the loaded condition, but no significant difference was found between those of hemoglobin oxygenation index values of MF and sham exposures at 5-min intervals (Fig 4B).

Fig 5: Effects of MF exposure on ECG parameters

The repeated-measures ANOVA of RRI showed no significant Condition by Time interaction [*F*(2, 36) = 0.51, *p* = 0.6038]. In the sham exposure, the RRI values have a tendency to be reduced during the motionless condition, but the RRI values were not significantly changed from the baseline value (indicated as 100%) (Fig 5A). In the MF-exposure, the RRI values were not significantly changed from the baseline value (Fig 5A). Therefore, there was no significant difference between both conditions (Fig 5A).

In the HF values, the repeated-measures ANOVA showed a significant Condition by Time interaction [*F*(50, 900) = 1.67, *p* = 0.0030]. The MF exposure significantly increased HF component values during the exposure and after the termination of the exposure periods from the baseline value (indicated as 100%) and the HF value peaked at 14-min exposure from the baseline value by 74% (Fig 5B). With regard to the differences between the MF and sham exposures, the HF values were significantly increased by the MF exposure compared with the sham exposure intermittently during the exposure and after the termination of the exposure periods (Fig 5B).

In the MF and sham exposures, the LF/HF ratio values have a tendency to be increased from the baseline values (indicated as 100%) under both conditions (Fig 5C). However, the repeated-measures ANOVA showed no significant Condition by Time interaction [*F*(50, 900) = 0.67, *p* = 0.9608]. No significant change in LF/HF ratio values was induced by the MF exposure, and there was no significant difference between the MF and sham exposures (Fig 5C).

Fig 6: Effects of MF exposure on FMD

The FMD values were evaluated before (baseline) and after 30-min exposure to MFs as described previously [51]. The repeated-measures ANOVA showed a significant Condition by Time interaction [*F*(1, 30) = 11.98, *p* = 0.0016]. The FMD values were signiﬁcantly increased from the baseline value in the presence of MF exposure (Fig 6). There were signiﬁcant differences between the MF and sham exposures (Fig 6).
